# Deciphering the Mto electron uptake pathway of *Sideroxydans lithotrophicus* ES-1

**DOI:** 10.1128/aem.02536-25

**Published:** 2026-06-02

**Authors:** Anaísa Coelho, Büşra Bayar, Bruno M. Fonseca, Abhiney Jain, Smilja Todorovic, Filipe Folgosa, Jeffrey A. Gralnick, Ricardo O. Louro, Catarina M. Paquete

**Affiliations:** 1 Instituto de Tecnologia Química e Biológica António Xavier, Universidade Nova de Lisboa 98819 , Oeiras, Portugal; 2 BioTechnology Institute and Department of Plant and Microbial Biology, University of Minnesota—Twin Cities 172728, St. Paul, Minnesota, USA; Michigan State University, East Lansing, Michigan, USA

**Keywords:** Fe(II)-oxidizing bacteria, extracellular electron transfer, multiheme cytochromes, electron uptake pathway

## Abstract

The freshwater, chemolithoautotrophic, Gram-negative bacterium *Sideroxydans lithotrophicus* ES-1 (ES-1) is thought to couple extracellular electron transfer (EET) to energy conservation through the Mto pathway and additional porin-cytochrome complexes, including Cyc2. The overall architecture and flexibility of ES-1 EET pathways remain poorly understood. Here, we investigated key components of the Mto pathway, focusing on the roles and interactions of the monoheme cytochromes MtoD and Slit_2494 (here designated as Periplasmic monoheme cytochrome A, PmcA), together with the inner-membrane tetraheme cytochrome ImoA. We performed the first biochemical and spectroscopic characterization of ImoA and PmcA, and investigated the interactions between multiple Mto pathway components using a combination of *in vitro* and *in vivo* approaches. We demonstrated that PmcA, but not MtoD, interacts with ImoA, and further show that PmcA, unlike MtoD, also interacts with MtoA. Our results identify PmcA as the physiological redox partner of ImoA, supported by favorable thermodynamics and direct protein-protein interactions. In contrast, the high redox potential of MtoD renders electron transfer to ImoA thermodynamically unfavorable. Instead, MtoD may transfer electrons to a high-potential downstream acceptor, such as a terminal oxidase. These findings refine and revise the previously proposed EET model for ES-1 and clarify the distinct roles of periplasmic cytochromes within the Mto pathway.

**IMPORTANCE:**

Fe(II)-oxidizing bacteria play an important role in the biogeochemical cycling of iron, representing a promising class of organisms for the development of novel biotechnological processes, including bioelectrosynthesis. These organisms perform extracellular electron transfer (EET) by acquiring electrons generated from the oxidation of Fe(II) outside the cell and transferring them into their internal metabolic processes. The understanding of EET pathways of these organisms is critical for harnessing and engineering their metabolic capabilities. In this study, we characterized biochemically and spectroscopically the cytochromes ImoA and PmcA from the Gram-negative Fe(II)-oxidizing bacterium *Sideroxydans lithotrophicus* ES-1 (ES-1). Through *in vitro* and *in vivo* interaction studies, we identified a direct interaction between ImoA and PmcA, but not with MtoD, which had previously been proposed as the physiological redox partner of ImoA within the Mto pathway. Furthermore, while MtoA did not interact with MtoD, it did show an interaction with PmcA, suggesting that PmcA may serve as an electron shuttle between outer and inner membrane components. These findings revise the current model of the Mto pathway and reveal a versatile electron transfer network in ES-1. This work provides critical insights into the electron transfer architecture of neutrophilic Fe(II)-oxidizing bacteria and broadens our understanding of EET strategies that can be leveraged for bioelectrosynthesis in microbial electrochemical technologies.

## INTRODUCTION

Neutrophilic chemolithoautotrophic Fe(II)-oxidizing bacteria thrive in different environments, including freshwater, marine, and subsurface habitats, playing important roles in the biogeochemical cycles of iron, carbon, nitrogen, phosphorus, and other metals (1–3). One example of these bacteria is the Gallionellacea *Sideroxydans lithotrophicus* ES-1 (ES-1), which is an Fe(II)-oxidizing bacteria, being able to oxidize Fe(II) or thiosulfate while reducing oxygen to fix carbon dioxide (4–6). As Fe(III) is insoluble under the physiological conditions of ES-1, Fe(II) oxidation must occur outside of the cell to prevent intracellular precipitation (7). By performing extracellular electron transfer (EET), these organisms capture electrons from Fe(II) outside of the cell and direct them into their metabolic pathways (8), thus preventing intracellular Fe(III) accumulation and its toxic effects. This process is carried out directly by cell-surface or periplasmic proteins that have access to the extracellular environment through outer-membrane porin (9–11). According to transcriptomic and genomic studies, ES-1 is proposed to use two types of iron oxidases to uptake electrons from Fe(II) compounds: the monoheme cytochrome porin Cyc2 and porin-cytochrome complexes (5, 6, 12). ES-1 encodes three adjacent *cyc2* genes that are among the most highly expressed genes during Fe(II) oxidation (5, 6, 13). The Cyc2 protein functions as a porin containing two possible iron-binding sites and an N-terminal *c*-type heme, which facilitates electron transfer across the outer membrane to the periplasmic side (14). The best-characterized porin cytochrome complex in ES-1 is MtoAB, which consists of the decaheme *c*-type cytochrome MtoA and the porin-like protein MtoB. Based on homology, MtoA is proposed to be inserted within the MtoB porin, forming a conduit for EET (12, 15). MtoAB shares homology with the PioAB complex of *Rhodopseudomonas palustris* TIE-1, where PioA is also proposed to uptake electrons from extracellular Fe(II) (12, 16–18). These complexes are also related to the MtrAB system of *Shewanella oneidensis* MR-1, in which MtrA instead transfers electrons across the outer membrane to facilitate Fe(III) reduction (4, 19).

**Fig 1:**
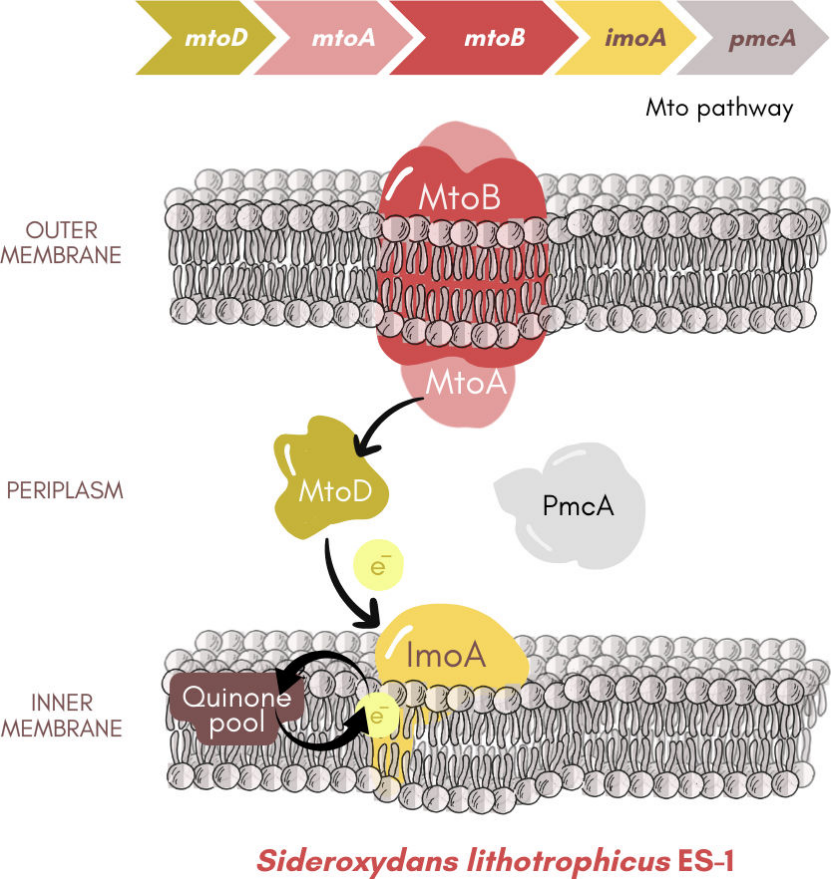
Gene cluster schematic and previously proposed model of the Mto pathway in ES-1. The model includes the cytochromes ImoA, MtoD, PmcA, and the porin cytochrome MtoAB.

MtoA can oxidize Fe(II) in the pH range of 7–9, and its hemes are redox active in the range between approximately +100 and −400 mV vs standard hydrogen electrode (SHE) (12). The *mtoA* and *mtoB* genes are located in a cluster encoded by *slit_2494-2498* on the ES-1 genome. This cluster, known as the Mto cluster, also comprises the genes *mtoD*, *imoA*, and *slit_2494,* suggesting that their protein products may work collectively to facilitate electron transfer across the cell envelope (12, 20). It was previously proposed that the monoheme cytochrome MtoD mediates electron transfer in the periplasmic space of ES-1 (Fig. 1), potentially serving as the physiological partner of the inner-membrane oxidoreductase tetraheme cytochrome ImoA and MtoA (21). Its heme group has a reduction potential of +155 mV vs SHE at pH 7, which makes it thermodynamically capable of receiving electrons from MtoA. However, this potential is significantly higher than that of menaquinol (−80 mV vs SHE) and ubiquinol (+100 mV vs SHE), raising doubts about whether MtoD can effectively transfer electrons to these quinones (21). Transcriptomic studies provide limited support for the role of MtoD in Fe(II)-dependent EET. While expression of *mtoD* was observed in Fe(II)-oxidizing cultures in the work (5), it was not detected under the conditions tested in (6). Instead, the monoheme cytochrome Slit_2494, encoded within the Mto gene cluster, exhibited expression patterns similar to those of other Mto-related proteins (6). These findings suggest that Slit_2494 may play a more prominent role in EET during Fe(II) oxidation in ES-1 than previously recognized.

To further elucidate the individual roles of Mto pathway components, we produced and biochemically and spectroscopically characterized the cytochromes ImoA and Slit_2494, the latter of which we designated periplasmic monoheme cytochrome A (PmcA). Additionally, we investigated their interactions with proposed physiological partners within the Mto pathway, using both *in vitro* and *in vivo* approaches. These studies were designed to clarify the molecular mechanisms of extracellular electron uptake employed by ES-1 during Fe(II) oxidation. Our results demonstrate that ES-1 possesses a more versatile and interconnected electron transfer network than previously documented, in which ImoA plays a central role by interacting with PmcA but not with MtoD. This work reveals a complex EET architecture in ES-1, broadening our understanding of Fe(II)-oxidation metabolism and highlighting their roles in the iron biogeochemical cycle and bioelectrosynthesis within microbial electrochemical technologies.

## RESULTS

### ImoA is a tetraheme cytochrome with one high-spin and three low-spin hemes

**Fig 2:**
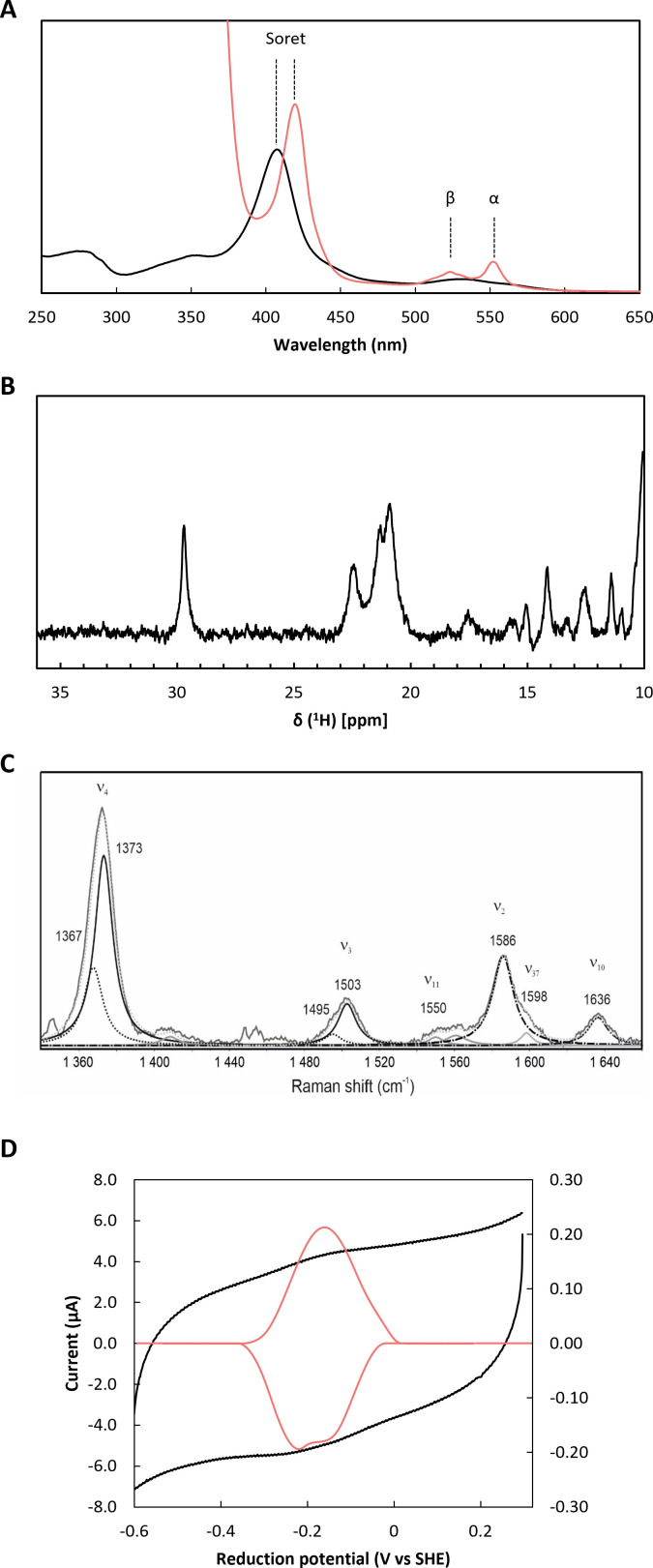
Biochemical properties of ImoA. (**A**) UV-visible spectra obtained in the oxidized (black) and reduced state (red); (**B**) ^1^H-1D-NMR spectrum obtained in the oxidized state; (**C**) high-frequency region of RR spectra. Experimental spectrum (light gray, solid line) and overall deconvoluted spectrum (light gray, dotted line), with designated ν_4_ and ν_3_ high-spin (black dotted trace) and low-spin (black solid trace) bands, non-assigned and spin-state non-specific bands (gray solid trace) and spin-state specific, non-deconvoluted bands ν_2_ and ν_10_ (black dot-dashed trace); (**D**) cyclic voltammetry data showing raw current (black and left axis) and baseline-subtracted data (red and right axis) obtained at 400 mV/s at pH 7.0.

Pure ImoA migrated as a single band in the SDS-PAGE gel, staining positively for covalently attached hemes (Fig. S1), and presented an absorbance ratio of A_Soret Peak_/A_280nm_ of 3.5 (Fig. 2A). N-terminal sequencing confirmed the presence of the intact N-terminal sequence of ImoA (NNKTG), consistent with its anchoring to the inner membrane via an uncleaved transmembrane segment, a characteristic of NapC/NirT family proteins. Mass spectrometry revealed that the purified ImoA had a molecular mass of ~26.5 kDa. This molecular weight agrees with the calculated molecular mass of the apo-protein (22.9 kDa) with the incorporation of the four hemes (~0.6 kDa per heme) and the Strep-tag (1 kDa). Gel filtration of ImoA revealed an apparent molecular mass of approximately 80 kDa, consistent with the protein existing as a monomer within an *n*-dodecyl-β-D-maltoside (DDM) micelle (comprising 51 kDa DDM micelle and the 25.5 kDa ImoA).

UV-visible spectra of ImoA show the typical features of a low-spin *c*-type cytochrome, with the characteristic Soret peak in the oxidized protein with a maximum at 408 nm that upon reduction of ImoA shifts to 420 nm. In the reduced state of the protein, the α and β peaks appear with maxima at 552 and 523 nm, respectively (Fig. 2A). The ^1^H-1D-NMR spectrum of ImoA in the oxidized state exhibits the typical features of a cytochrome with low-spin paramagnetic hemes, with moderately sharp signals up to 40 ppm (22) (Fig. 2B). However, Resonance Raman (RR) spectroscopy revealed asymmetric and broadened heme spin and oxidation state marker bands (ν_i_), particularly in the ν_4_ and ν_3_ regions, suggesting the presence of two distinct ferric heme populations. Component analysis of the spectra enabled the precise determination of the frequency of the ν_4_ mode at 1,373 cm^−1^ and that of the ν_3_ at 1,503 cm^−1^ , which are characteristic of a low-spin state, and the frequency of the ν_4_ mode at 1,367 cm^−1^ and that of the ν_3_ at 1,495 cm^−1^ that are indicative of a high-spin population (Fig. 2C) (23, 24). The relative ratio of the band intensities (i.e., ν_3_ [LS]/ν_3_ [HS]), together with the fact that the protein contains four hemes, is consistent with ImoA having one high-spin and three low-spin hemes.

Based on sequence comparisons with other NapC/NirT family proteins, to which ImoA belongs, heme I is predicted to be the high-spin heme, typically coordinated by a methionine within the CXXCHXM motif (25). ImoA hemes II, III and IV are predicted to be low-spin bis-His axially coordinated by the conserved distal histidines His186, His74, and His167, respectively (Fig. S2). The methionine residue associated with heme I is fully conserved in NrfH proteins and in some NapC sequences (26), including ImoA, but it is absent in CymA (27). However, the NMR spectrum of reduced ImoA in the presence of a strong-field ligand (e.g., cyanide), which promotes octahedral coordination of the heme (Fig. S3), showed no signal near −3 ppm, the region where a methionine-coordinated heme would typically resonate. This observation suggests that in ImoA the histidine from the CXXCH motif is the proximal axial ligand of heme I, as is typical of *c*-type hemes (28, 29). Interestingly, the lysine residue (Lys 101), predicted to play a role in menaquinone interaction, electron transfer, and possible proton translocation in other NapC/NirT family proteins is conserved in ImoA (30).

The redox properties of ImoA were assessed by protein film voltammetry (Fig. 2D), revealing that the protein undergoes redox transitions between 0 and −400 mV over a pH range of 6 to 9. Notably, the pH-dependent changes observed in the voltammograms (Fig. S4) indicate the presence of redox-Bohr effect (31). However, the individual reduction potentials of the hemes of ImoA remain unknown, as the discrimination of the hemes in this protein has not yet been performed (32).

### ImoA exhibits a mode of action distinct from CymA

**Fig 3:**
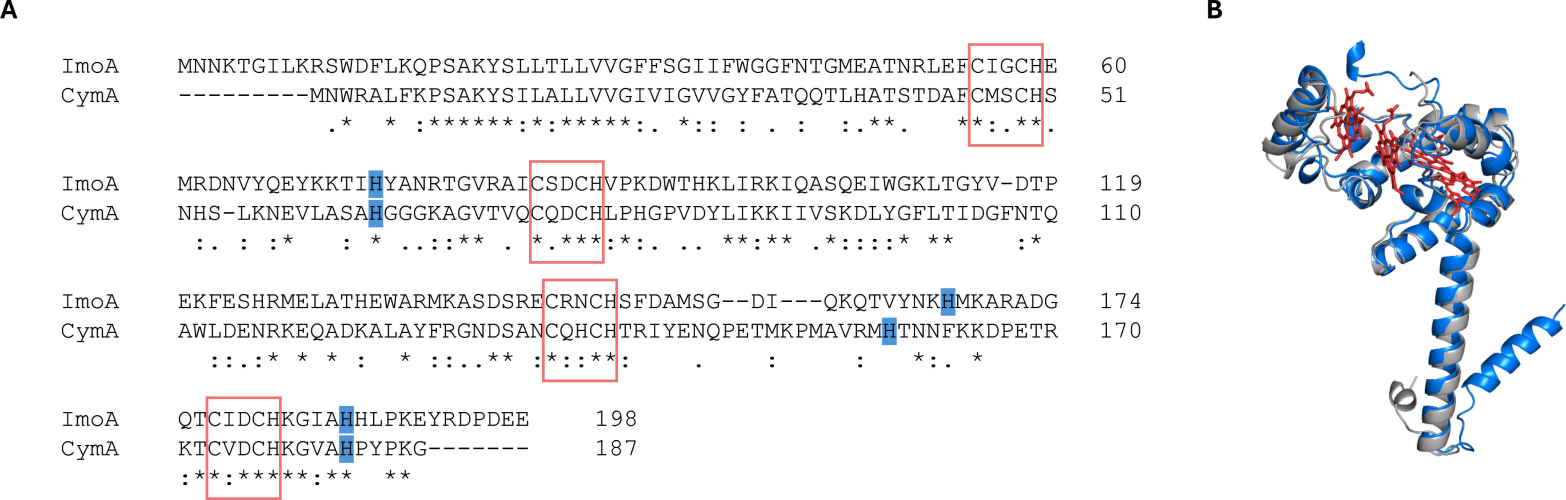
Comparison of ImoA from ES-1 and CymA from *S. oneidensis* MR-1. (**A**) Sequence alignment of ImoA and CymA using ClustalW. The heme-binding motifs are shown in red boxes. The distal histidine of the low-spin hemes is highlighted in blue. (**B**) Predicted model of the three-dimensional structure of ImoA (in blue) using Alphafold3 and comparison with that of CymA (in light gray). The hemes colored in red are of ImoA.

ImoA, like CymA from *S. oneidensis* MR-1, is a tetraheme cytochrome consisting of an N-terminal α-helical domain, and a C-terminal globular domain that contains the four heme groups. Despite sharing only 33% sequence identity (33), the two proteins display highly similar predicted three-dimensional structures, with conserved heme-binding motifs and well-aligned heme positions (Fig. 3).

This predicted structural similarity, along with the observation that ImoA can functionally replace CymA in oxidizing the menaquinone pool of *S. oneidensis* MR-1 (33), suggests that both proteins may operate through similar mechanisms. To further investigate this possibility, interaction studies followed by NMR spectroscopy were conducted between ImoA and the native redox partners of CymA in *Shewanella*, STC, and FccA (Fig. S5). These studies enabled us to directly assess and compare the modes of action of ImoA and CymA. Perturbation in the NMR signals of heme methyl groups in both STC and FccA upon addition of ImoA indicates that these proteins interact in a slow exchange regime (34), consistent with a relevant interaction likely involved in electron transfer. Since the NMR signals of STC and FccA have been previously assigned to individual hemes (35, 36), it was possible to identify the specific interaction sites. STC interacts with ImoA via hemes II, III, and IV, while FccA interacts through heme I. In contrast to CymA, which engages STC exclusively via heme IV and FccA via heme II (37), ImoA follows a distinct interaction scheme, pointing to an alternative mechanism of action within the periplasmic space of *Shewanella*.

### PmcA is a monoheme cytochrome with distinct redox properties from MtoD

**Fig 4:**
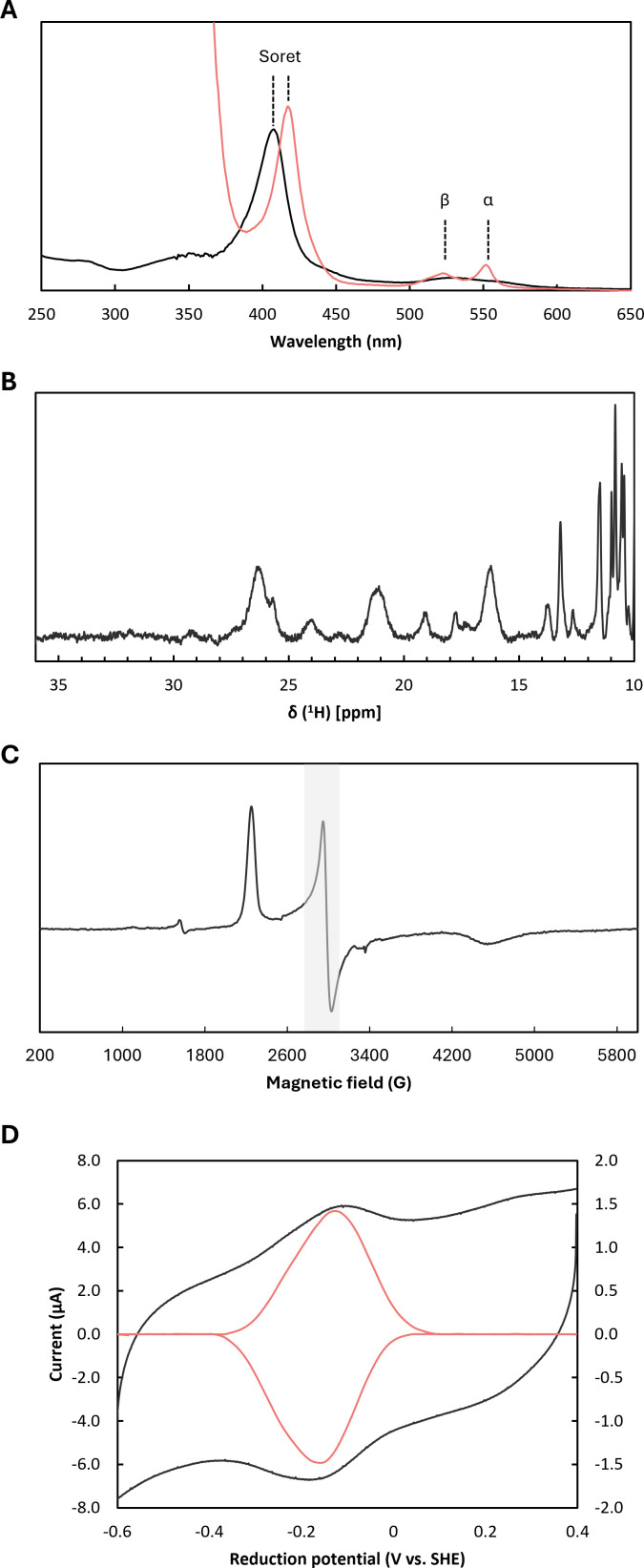
Biochemical properties of PmcA (**A**) UV-visible spectra obtained in the oxidized (black) and reduced state (red line); (**B**) ^1^H-1D-NMR spectrum obtained in the oxidized state; (**C**) EPR spectrum obtained in the oxidized state at 15K (black line) and fitted data (dashed line); (**D**) cyclic voltammetry data showing raw current (black and left axis) and baseline-subtracted data (red and right axis) obtained at 400 mV/s and pH 7.0.

Recombinant PmcA was successfully purified as a homogeneous protein, appearing as a single band of approximately 11 kDa in SDS-PAGE (Fig. S6), and presented an absorbance ratio of A_Soret Peak_/A_280nm_ of 5 (Fig. 4A). N-terminal sequencing confirmed the predicted sequence of the protein without the signal peptide (DVSFK), indicating that the protein was correctly processed and matured in *Escherichia coli*. Mass spectrometry further validated the expected molecular weight of 11.5 kDa, consistent with the presence of a covalently bound *c*-type heme. Size exclusion chromatography revealed that PmcA behaves as a monomer in solution.

Spectroscopic analysis confirmed that PmcA is a low-spin cytochrome *c*. The distinctive absorbance bands of *c*-type cytochrome were visible in the UV-visible spectra. In the oxidized state, the Soret peak was located at 408 nm (Fig. 4A), which shifts to 417 nm when PmcA was reduced. The bands α and β were also detected in the reduced state, with their respective maxima at 552 and 523 nm (Fig. 4A). The ^1^H-1D-NMR spectrum of the oxidized state exhibits signals corresponding to heme methyl groups in the low-field region (Fig. 4B). Upon reduction, a characteristic upfield signal at −3 ppm was observed (Fig. S7), consistent with a methionine serving as the distal axial ligand to the heme (29). Methionine 74 can function as the axial ligand of the heme, as shown by the structure of PmcA predicted by Alphafold3 (Fig. S8). The EPR spectrum at 15K of the oxidized form of PmcA (Fig. 4C) revealed a rhombic low‐spin (S = 1/2) ferric heme signal characterized by *g*‐values of 2.98, 2.25, and 1.46. These values are consistent with a low‐spin heme, which is similar to that reported for other histidine-methionine coordinated heme proteins, such as the heme *c* of cytochrome *cd*₁ from *Pseudomonas stutzeri* and cytochrome *c*_555_ from *Methylococcus capsulatus* (38, 39). The absence of signals in the high-spin region (*g* ~6) confirms that the heme population is exclusively low-spin under these conditions.

Protein film voltammetry of PmcA revealed a reduction potential of approximately −143 mV (vs SHE) at pH 7.0, consistent with its classification as a low-potential cytochrome (Fig. 4D). PmcA exhibits the redox-Bohr effect, as evidenced by the pH-dependent shifts observed in its voltammograms (Fig. S9), with the heme reduction potential increasing to –97 mV as the pH decreases to pH 6.0 (39, 40).

### ImoA interacts with PmcA but not with MtoD

**Fig 5:**
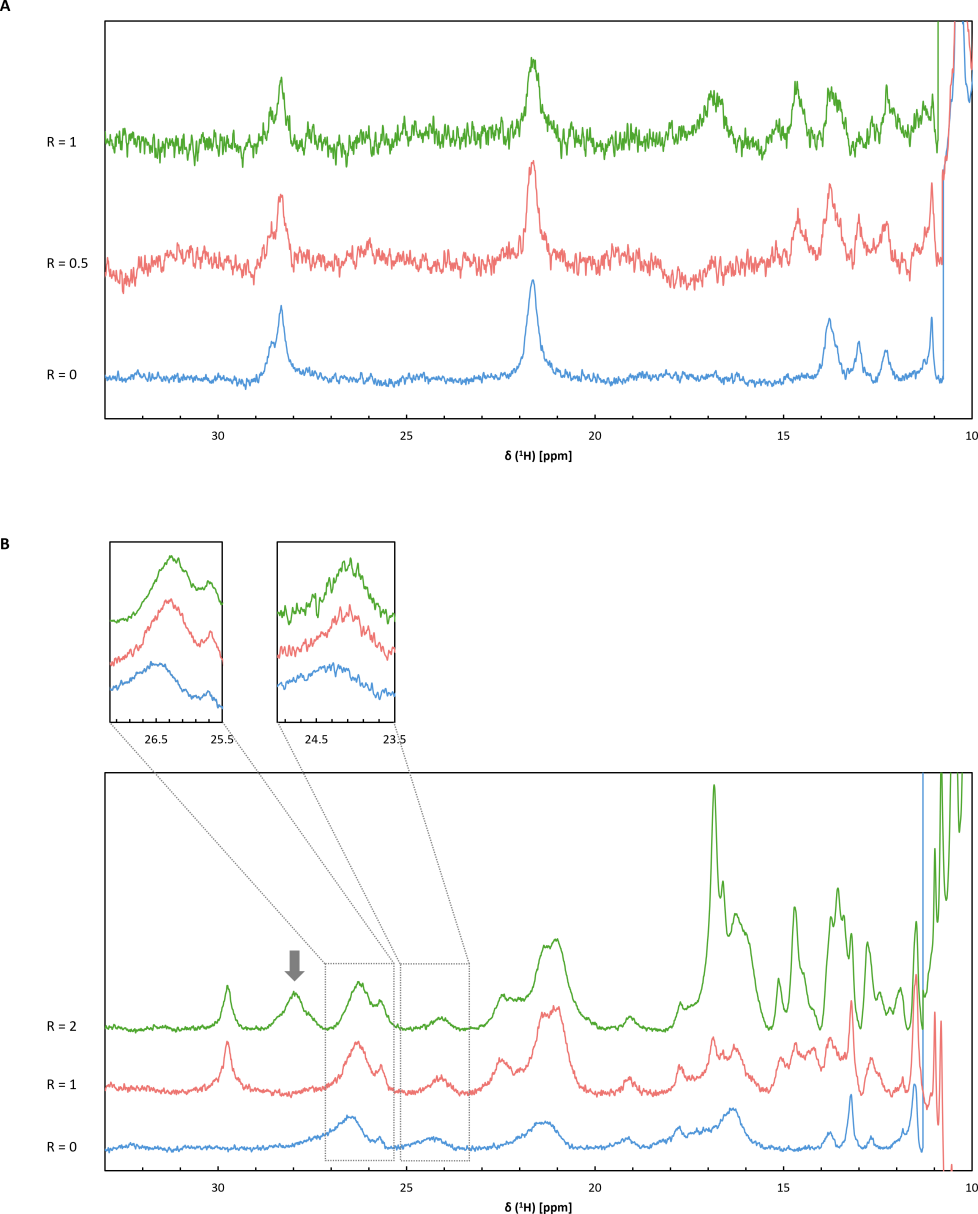
^1^H-1D-NMR spectral changes of (**A**) MtoD and (**B**) PmcA in the presence of ImoA. The bottom spectra were obtained with the individual monoheme protein in the oxidized state, while the top spectra were obtained upon increasing amounts of ImoA (molar ratio of [ImoA]/[MtoD or PmcA]). Gray boxes indicate spectral changes in the monoheme cytochrome upon addition of the ImoA protein, while arrows highlight features that shift in the spectrum of the added cytochrome.

To assess possible interactions between ImoA and the cytochromes PmcA and MtoD, we conducted NMR spectroscopy by monitoring the heme methyl group signals of these proteins as ImoA was gradually added. Such chemical shift changes are indicators of protein–protein interactions relevant for electron transfer (37, 41). For MtoD, no significant chemical shift changes were observed in the paramagnetic region of the spectra, where the heme methyl signals appear, with increasing ImoA concentrations (Fig. 5A). The only notable change was the appearance of two signals around 14.5 and 16.5 ppm, which belong to the ImoA protein. The lack of perturbation suggests that ImoA probably does not interact with MtoD, or if it does, the interaction does not involve the region around the heme and is unlikely to be relevant for electron transfer.

In contrast, titration of ImoA into PmcA led to clear chemical shift perturbation of the heme methyl signals of PmcA (Fig. 5B), along with the appearance of a new signal in the NMR spectrum. The appearance of this new signal is likely associated with ImoA and may reflect a conformational change or an altered heme environment upon interaction, resulting in spectral characteristics absent in the unbound state. This indicates a direct interaction between ImoA and PmcA, which likely occurs in the vicinity of the heme. Given that PmcA contains a single heme, this interaction is likely functionally relevant for electron transfer. Since the heme methyl signals have only been assigned for MtoD (42), it is not possible to determine the specific interaction site between ImoA and PmcA.

### PmcA, but not MtoD, facilitates electron transfer to electrodes in *Shewanella*

**Fig 6:**
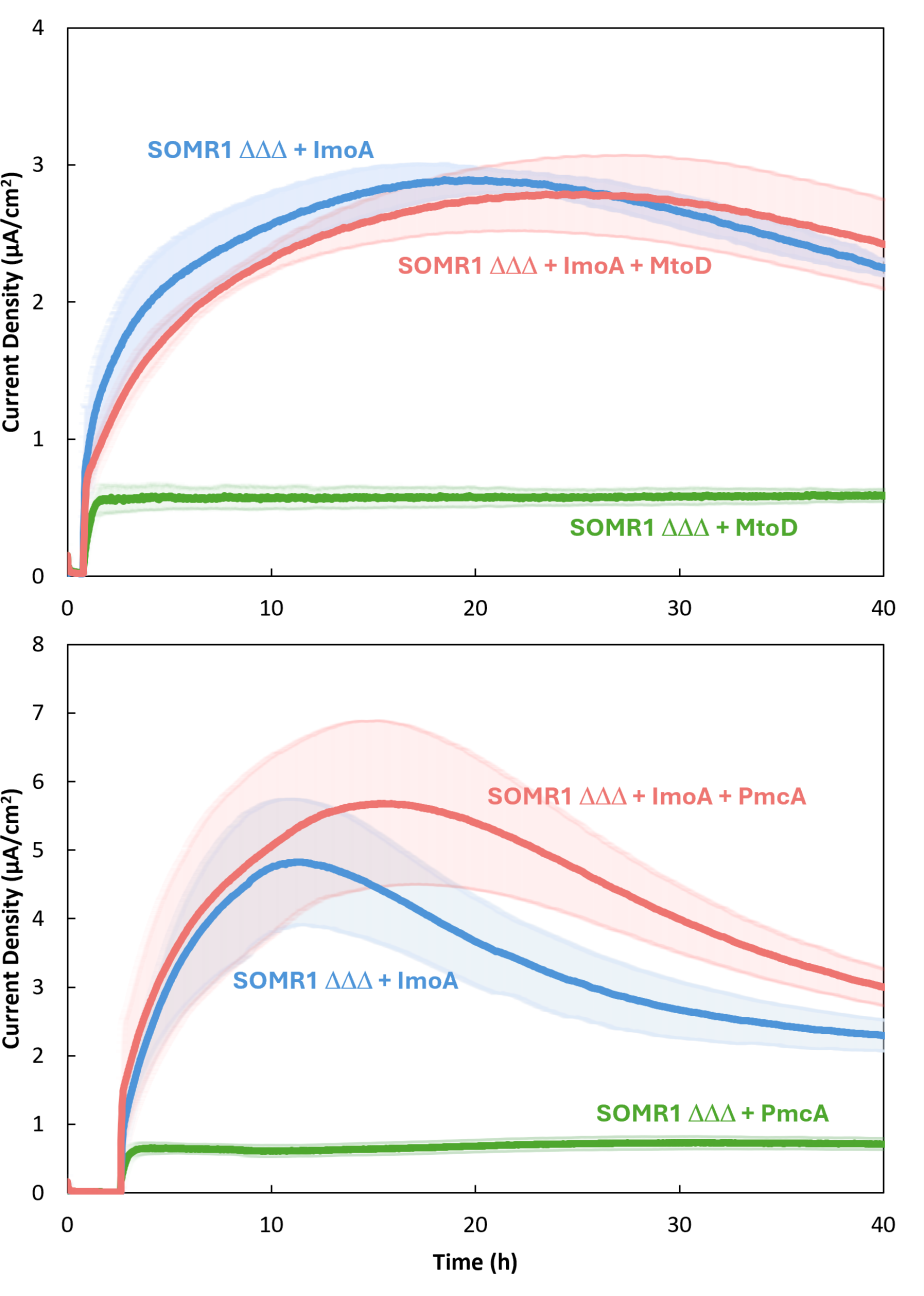
Current density produced by the different Δ*cymA* Δ*fccA* Δ*cctA Shewanella* strains. *Shewanella* Δ*cymA* Δ*fccA* Δ*cctA* strains containing pBBR1MCS-2::*imoA* (blue line), pBBR1MCS-2::*mtoD* or pBBR1MCS-2::*pmcA* (red line), pBBR1MCS-2::*imoA::mtoD* or pBBR1MCS-2::*imoA::pmcA* (red line) in bioelectrochemical reactors. Each panel represents an independent experiment, each with several replicates. Internal control strains were included in both to address batch-to-batch variability and variations in current outputs across experimental datasets. Error bars shown in the same colors represent the standard deviation of the mean from experiments performed in triplicates.

Since a genetic system has not yet been established for ES-1, the interaction between ImoA, MtoD, and PmcA was investigated through *in vivo* studies in *S. oneidensis*. In these experiments, ImoA, MtoD, and PmcA were heterologously expressed in *S. oneidensis* deletion mutant lacking CymA (Δ*cymA*), STC (Δ*cctA*), and FccA (Δ*fccA*) in different combinations. Their ability to support EET to an electrode was evaluated by measuring current production in a three-electrode bioelectrochemical reactor (Fig. 6).

While *Shewanella* Δ*cymA* Δ*fccA* Δ*cctA* strain harboring an empty plasmid, as well as the strains carrying only MtoD or PmcA produced negligible current (Fig. S10), strains expressing ImoA alone (*Shewanella* Δ*cymA* Δ*fccA* Δ*cctA* pBBR1MCS2::*imoA*), ImoA with PmcA (*Shewanella* Δ*cymA* Δ*fccA* Δ*cctA* pBBR1MCS2::*imoA::pmcA*), or ImoA with MtoD (*Shewanella* Δ*cymA* Δ*fccA* Δ*cctA* pBBR1MCS2::*imoA::mtoD*) generated significant levels of current. These results confirm that ImoA is required for *Shewanella* to perform EET in this genetic background, facilitating electron transfer from the menaquinone pool to periplasmic cytochromes, consistent with our previous findings (33).

Co-expression of PmcA with ImoA resulted in a minor increase in current generation, whereas expression of MtoD did not result in a measurable change. These observations suggest that under the conditions tested, PmcA has a more pronounced effect on EET than MtoD.

### MtoA interacts with PmcA but not with MtoD

**Fig 7:**
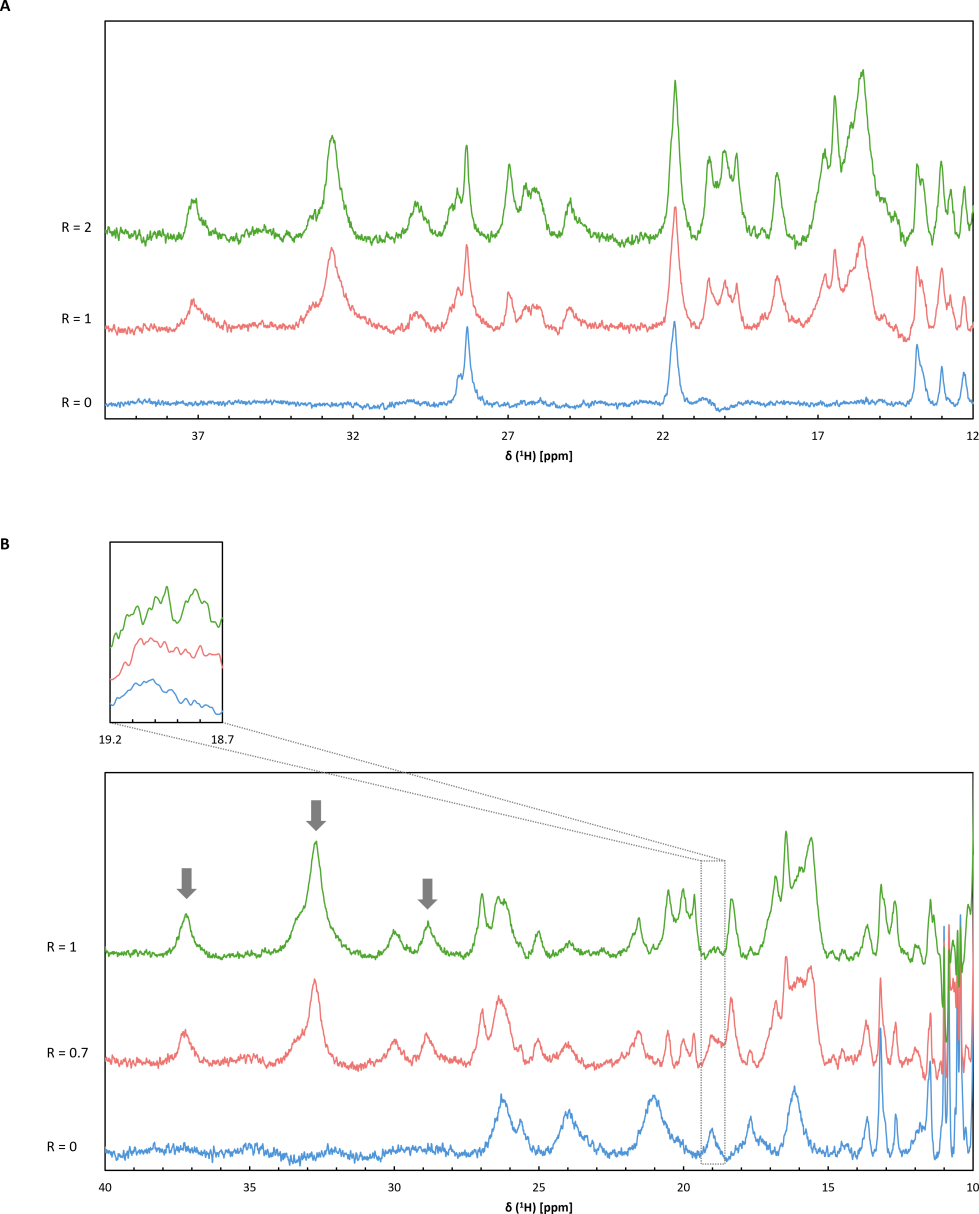
^1^H-1D-NMR spectral changes of (**A**) MtoD and (**B**) PmcA in the presence of MtoA. The bottom spectra were obtained with the individual monoheme protein in the oxidized state, while the top spectra were obtained upon increasing amounts of MtoA (molar ratio of [MtoA]/[MtoD or PmcA]). Gray boxes indicate spectral changes in the monoheme cytochrome upon addition of the MtoA protein, while arrows highlight features that shift in the spectrum of the added cytochrome.

To investigate the physiological partners of MtoD and PmcA, NMR interaction studies monitored by NMR spectroscopy were also conducted with the decaheme cytochrome MtoA (Fig. 7). The NMR spectra of MtoA show signals in the paramagnetic region, consistent with the presence of low-spin hemes, as expected based on homology of MtoA with other decaheme cytochromes from porin-cytochrome complexes (12, 42).

Titration of MtoD with MtoA did not result in any chemical shift perturbation in the paramagnetic region of the spectra (Fig. 7A). The only noticeable difference was the appearance of MtoA signals, which remained unchanged throughout the titration. The absence of spectral perturbation suggests that MtoA likely does not interact with MtoD. If any interaction does occur, it does not involve regions relevant for electron transfer.

In contrast, titration of MtoA into PmcA led to subtle chemical shift changes in the heme methyl signals of the PmcA spectrum (Fig. 7B), along with the appearance of additional signals. Although it is not possible to follow the chemical shifts of most PmcA resonances due to overlap with MtoA signals, the addition of MtoA induces a chemical shift in the isolated PmcA peak at 19 ppm. The newly appearing signals are attributed to MtoA and may reflect a modified heme environment upon binding, as MtoA signals shift during the titration, suggesting an interaction between the two proteins. To further validate these findings, we performed complementary NMR experiments by titrating MtoA with PmcA. The resulting spectra (Fig. S11) revealed chemical shift changes in the heme methyl signals of MtoA, confirming the interaction between the two proteins.

## DISCUSSION

Current understanding of the metabolic processes of ES-1 is largely derived from genomic analysis (4, 19), transcriptomics studies (5, 6), and heterologous expression (33, 43). Although proteins have been identified to be involved in EET processes, the electron transfer processes among the key players of the Mto pathway remain to be elucidated. Furthermore, while MtoA and MtoD cytochromes have undergone biochemical characterization (12, 21), ImoA and PmcA had not been biochemically characterized until now.

Biochemical characterization of ImoA revealed a redox potential range of 0 to −400 mV vs SHE, indicating that it is well-suited to exchange electrons from low-potential quinol pools such as menaquinones (44). Previous studies have shown that ImoA can oxidize quinol in the inner membrane of *S. oneidensis* and can functionally replace CymA (33). This capability likely arises from ImoA’s promiscuous interactions with native *Shewanella* periplasmic cytochromes, which explains the baseline current observed in strains expressing ImoA alone (Fig. 6). Nevertheless, the ES-1 genome lacks genes for menaquinone biosynthesis, which are typically required for anaerobic electron transport chains (45). Instead, it contains a complete set of genes for ubiquinone biosynthesis (Table S1). These findings suggest that, despite their structural similarity, ImoA and CymA operate differently, likely reflecting differences in their physiological roles (e.g., CymA oxidizes the quinone/quinol pool, while ImoA was proposed to reduce it in ES-1), or in the electron transfer partners they engage. Notably, ImoA interacts with STC and FccA through distinct hemes compared with CymA, pointing to a different mode of electron transfer. While speculative, this distinct interaction pattern of ImoA suggests a potential functional versatility, in which the protein may support electron flow in more than one direction, depending on its electron transfer partners and the metabolic conditions of the cell.

In ES-1, MtoD was initially considered the likely electron donor to ImoA due to their co-localization within the same gene cluster; however, its redox potential (+155 mV vs SHE) is too high to facilitate electron transfer to ImoA. In contrast, PmcA, with a midpoint redox potential of approximately −143 mV vs SHE, is better suited to serve as an electron donor to ImoA. These results are supported by interaction studies, followed by NMR experiments, which showed shifts in the heme methyl signal of PmcA, but not of MtoD, upon increasing amounts of ImoA. Interestingly, similar results were obtained with the proposed outer-membrane physiological partner MtoA. While PmcA was shown to interact with MtoA, MtoD did not, suggesting that MtoD does not participate in EET under the conditions tested. This lack of interaction implies a specific physiological role for PmcA in the Mto pathway. Importantly, the absence of interaction between ImoA and MtoD is not attributable to protein misfolding or inactivity. The purified MtoD protein exhibits UV-visible (Fig. S12) and NMR spectral features characteristic (Fig. 5A and 7A) of a correctly folded low-spin cytochrome (42). Furthermore, heterologous *in vivo* assays using a *S. oneidensis* ΔFccA ΔSTC strain demonstrated that both PmcA and MtoD can receive electrons from CymA and transfer them to MtrA (Fig. S13). Taken together, these findings are consistent with those found in the work (6), who reported that MtoD was not detected when ES-1 was grown on Fe(II)-containing solid minerals. Although the limitations of proteomic detection must be considered, the absence of a biochemical interaction between ImoA and MtoD observed in our study provides direct evidence supporting the conclusion that, under these conditions, PmcA, rather than MtoD, serves as the functional electron transfer partner of ImoA.

**Fig 8:**
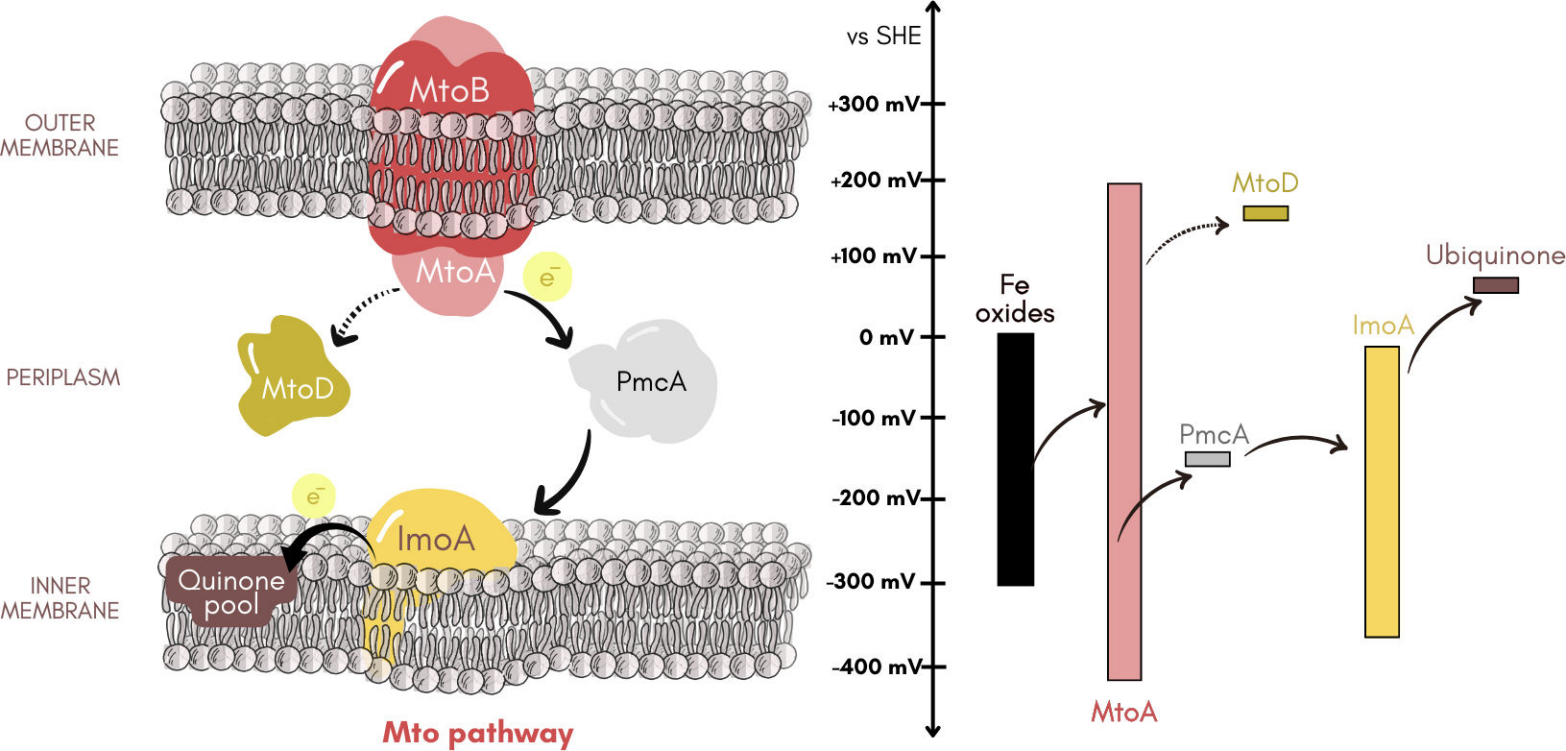
Revised model of the Mto pathway in ES-1. The schematic illustrates the cytochromes of the Mto pathway arranged along a redox ladder, incorporating the redox potentials of PmcA and ImoA determined in this study, together with previously reported values. Based on the biochemical and interaction data presented here, PmcA is identified as the physiological redox partner of ImoA, facilitating thermodynamically favorable electron transfer. In contrast, the high redox potential of MtoD suggests it directs electrons toward a high-potential downstream acceptor, such as a terminal oxidase.

Although MtoD and PmcA are localized in the same cellular compartment, NMR analysis revealed that these proteins do not interact with each other (Fig. S14). This is not unusual in EET pathways. For example, in *S. oneidensis* MR-1, the tetraheme cytochromes STC and FccA also do not interact directly, instead performing complementary roles (37). However, in ES-1, it is unlikely that MtoD and PmcA serve complementary functions, as they exhibit distinct biochemical properties, including distinct reduction potential, suggesting they participate in separate electron transfer routes rather than functioning together within the same pathway. Moreover, the genome of ES-1 encodes seven additional Mto-related monoheme cytochromes (Table S2), many of which are highly transcribed during iron oxidation and could contribute to electron transfer within the periplasm of ES-1 (5, 6). Importantly, none of these cytochromes cluster with the proteins from the Mto pathway, suggesting that they may perform distinct roles in periplasmic electron transfer. Notably, MtoD is unique among them, exhibiting bis-His axial coordination of its heme, whereas the others possess His–Met coordination, further highlighting its distinct functional role. The high redox potential of MtoD makes electron transfer to ImoA thermodynamically unfavorable (Fig. 8). Instead, we propose that MtoD is likely to transfer electrons to a higher-potential downstream acceptor, such as a terminal oxidase. This suggests that the *mto* cluster may encode a versatile electron transfer pathway, capable of channeling electrons from extracellular metals toward distinct intracellular acceptors: either to low-potential carriers (via PmcA) or to oxygen (via MtoD), depending on environmental conditions. This work highlights that, while genetic approaches can offer valuable insights into respiratory processes, a comprehensive understanding requires the integration of biochemical characterization and interaction studies. We are now at a point where combining multiple techniques and methodologies is crucial to unravel how microbes conduct electron transfer and, more importantly, how they can switch between diverse electron donors and acceptors. This metabolic flexibility not only enables their survival across a wide range of environments but also has significant implications for their roles in global biogeochemical cycles and for biotechnological applications where such capabilities can be harnessed.

## MATERIALS AND METHODS

### Construction of bacterial strains

The bacterial strains used in this study are listed in Table 1. Plasmids and primers are listed in Table S3. The *S. oneidensis* double-deletion mutant JG3107, lacking both STC and FccA, was generated using the Δ*cctA* strain (46) as the parental background. The plasmid was generated by amplifying approximately 1-kb DNA fragments upstream and downstream of the STC gene, including nine nucleotides after the start codon and nine nucleotides before the stop codon, followed by ligating and cloning into the suicide vector pSMV3 (47), which was transformed into chemically competent *E. coli* WM3064 cells (48, 49). Transformed *E. coli* WM3064 cells were selected on Lysogeny Broth (LB) medium plates containing 50 µg/mL kanamycin and 360 µg/mL diaminopimelic acid (DAP) and used to conjugate deletion constructs into desired *S. oneidensis* strains. Merodiploids were selected by growth on LB plates containing 50 µg/mL kanamycin without DAP. Merodiploids were resolved by sucrose counterselection mediated by SacB and screened by PCR.

**TABLE 1:** Bacterial strains used in this study

Bacterial strain	Source
Deletion strains	
*Shewanella oneidensis* Δ*cymA* Δ*fccA* Δ*cctA* (JG3745)	Gralnick Lab Strains Collection (50)
*Shewanella oneidensis* Δ*fccA* Δ*cctA* (JG3107)	This study
*Shewanella oneidensis* Δ*omcs* (JG132)	(51)
Expression strains	
*Escherichia coli* JM109(DE3) containing pEC86	IBN Lab Strains Collection
*Escherichia coli* JM109(DE3) containing pEC86 and pBAD*::imoA*	This study
*Escherichia coli* JM109(DE3) containing pEC86 and pBAD*::pmcA*	This study
*Escherichia coli* JM109(DE3) containing pEC86 and pBAD*::mtoD*	(42)
*Shewanella oneidensis* Δ*omcs* containing pBAD*::mtoA*	This study
Strains for phenotype studies	
Δ*cymA* Δ*fccA* Δ*cctA* containing pBBR1MCS-2::*imoA*	(33)
Δ*cymA* Δ*fccA* Δ*cctA* containing pBBR1MCS-2::*pmcA*	This study
Δ*cymA* Δ*fccA* Δ*cctA* containing pBBR1MCS-2::*mtoD*	This study
Δ*cymA* Δ*fccA* Δ*cctA* containing pBBR1MCS-2::*imoA::mtoD*	This study
Δ*cymA* Δ*fccA* Δ*cctA* containing pBBR1MCS-2:: *imoA::pmcA*	This study
Δ*fccA* Δ*cctA* containing pBBR1MCS-2::*pmcA*	This study
Δ*fccA* Δ*cctA* containing pBBR1MCS-2::*mtoD*	This study

The construction of heterologous expression strains (Table 1) was carried out by cloning the key genes into the NcoI restriction site of the pBAD202/D-TOPO plasmid (Invitrogen, Carlsbad, CA, USA) using the NEBuilder Assembly kit (New England BioLabs). The gene of *imoA* was synthesized by NZYTech, retaining its native N-terminal transmembrane segment, while the gene *pmcA* was synthesized by Eurofins Genomics, with its native signal peptide replaced by the signal peptide of OmpA from *E. coli* to ensure efficient periplasmic translocation in *E. coli* (42). The gene *mtoA* was obtained from the *mto* cluster previously cloned in pBBR1MCS-2 (43). The sequence of the signal peptide of MtoA was also replaced by that of OmpA from *E. coli* using the strategy previously described (42). The DNA fragment coding the signal peptide of OmpA was amplified from the genomic DNA of *E. coli*. To facilitate the purification process, a Strep-tag sequence was included in the reverse primer before the stop codon of the genes *imoA* and *mtoA*. The plasmids pBAD202::*imoA_strep* and pBAD202::*sp-ompA_pmcA* were transformed into chemically competent *E. coli* strain JM109(DE3) co-transformed with vector pEC86 containing the *ccmABCDEFGH c*-type maturation genes (52), while plasmid pBAD202::*mtoA* was transformed into *S. oneidensis*Δ*omcs* lacking native outer-membrane multiheme cytochromes (51).

For the phenotype studies, the *imoA*, *pmcA,* and *mtoD* genes were amplified by PCR and cloned into pBBR1MCS-2 vector (53) using the NEBuilder Assembly kit (New England BioLabs) using the primers listed in Table S3. The dual-gene constructs were performed for the gene pairs *imoA* with *mtoD*, and *imoA* with *pmcA*, using the phusion assembly strategy, and inserted into the same vector, also with NEBuilder Assembly kit (New England BioLabs). pBBR1MCS-2 was a gift from Kenneth Peterson (Addgene plasmid # 85168; https://www.addgene.org/85168/; RRID:Addgene_85168). The resulting plasmids (Table S3) were then transformed into *Shewanella* using electroporation (54).

### Protein production

#### Production of ImoA

The *E. coli* strains JM109 (DE3) harboring pEC86 and pBAD202::*imoA* were grown aerobically at 37°C in Terrific Broth (TB) medium containing 50 µg/mL kanamycin and 34 µg/mL chloramphenicol in 5-L Erlenmeyer flasks containing 2 L of medium at 150 rev./min. Protein expression was induced by the addition of 3 mM L-arabinose at an optical density (OD_600nm_) of 1.0. Cells were allowed to grow under the same conditions for 48 h before harvesting. Bacterial cells were harvested by centrifugation at 10,000 × *g* for 15 min at 4°C. The cell pellet collected from 6-L growth was resuspended in 20 mM potassium phosphate buffer (pH 7.6) containing protease inhibitor cocktail (Roche), and DNase I (Sigma). The disruption of the cells was achieved by three passages through a French Press at a pressure of 1,000 psi (6.89 MPa). The crude extract was centrifuged at 200,000 g for 1 h at 4°C (Beckman Coulter Optima LE-80K). To solubilize the membrane proteins, the membrane pellet was homogenized and solubilized in 20 mM potassium phosphate buffer (pH 7.6) with 300 mM NaCl, containing a protease inhibitor cocktail and 4% (w/v) DDM at 4°C overnight. The insoluble material was removed by ultracentrifugation at 200,000 × *g* for 1 h at 4°C. Harvested clear cell lysates containing the solubilized membrane proteins were loaded into a 5 mL Strep-Tactin column (IBA), and the protein was eluted using Elution Buffer (20 mM potassium phosphate buffer (pH 7.6), 300 mM NaCl, 0.05% DDM, 2.5 mM desthiobiotin). Eluted fractions were analyzed by SDS-PAGE (12% gel) stained for heme proteins (55) and by UV-visible spectroscopy to select those containing pure protein, measured by the A_Soret peak_/A_280nm_ ratio. To provide direct insight into the oligomeric state of ImoA, pure protein was concentrated and subjected to size-exclusion chromatography on a Superdex 200 10/300 GL column (GE Healthcare) in 20 mM potassium phosphate buffer (pH 7.6), 300 mM NaCl containing 0.05% DDM. The identity of ImoA was confirmed by N-terminal sequencing and mass spectrometry.

#### Production of MtoA

The production of MtoA was performed in *S. oneidensis* MR-1 transformed with the inducible pBAD202 containing MtoA with a strep tag (pBAD*::mtoA*). The cultures were grown aerobically at 37°C in TB medium containing 50 µg/mL kanamycin, using 5-L Erlenmeyer flasks filled with 2 L of TB medium and agitated at 150 rev./min. Expression of MtoA was induced with 3 mM L-arabinose when the cultures reached an OD₆₀₀ of 0.6, followed by continued incubation for 4 h under the same conditions. Cells from a total of 6-L culture were harvested by centrifugation at 10,000  ×  *g* for 15 min at 4°C. The cell pellet was resuspended in 20 mM HEPES buffer (pH 7.8) containing 150 mM NaCl, a protease inhibitor cocktail (Roche), and DNase I (Sigma). A French Press was used for cell disruption, with three passes at 1,000 psi (6.89 MPa). The lysate was clarified by ultracentrifugation at 200,000  ×  *g* for 1 h at 4°C using a Beckman Coulter Optima LE-80K. The resulting supernatant, containing soluble proteins, was loaded onto a 5 mL Strep-Tactin (Superflow) affinity column (IBA). Bound MtoA was eluted with a buffer containing 20 mM HEPES (pH 7.8), 150 mM NaCl, and 2.5 mM desthiobiotin. Eluted fractions were analyzed by 12% SDS-PAGE, followed by heme staining (55), and protein purity was further assessed by UV-visible spectroscopy based on the Soret peak to A₂₈₀ ratio of 6 (12). The identity of MtoA was confirmed by mass spectrometry.

#### Production of MtoD

The production of MtoD was carried out using the plasmid pBAD202:*:mtoD* in *E.coli* JM109(DE3) containing the plasmid pEC86, as previously described (42). Protein purity was confirmed by a single band on a 12% SDS-PAGE gel, along with an A_Soret peak_/A_280nm_ ratio of 5, as determined by UV-visible spectroscopy.

#### Production of PmcA

The production of PmcA was achieved using the *E.coli* JM109(DE3) containing pEC86 and pBAD::*sp-ompA_pmcA*, grown aerobically at 37°C in LB medium containing 50 µg/mL kanamycin and 34 µg/mL chloramphenicol in 5-L Erlenmeyer flasks containing 2 L of medium at 150 rev./min. The expression of the protein was carried out by the addition of 10 mM L-arabinose at an optical density (OD_600nm_) of 1.0. Cells were allowed to grow under the same conditions for 16 h before harvesting. Bacterial cells were harvested by centrifugation at 10,000 × *g* for 15 min at 4°C. The cell pellet from the 6-L growth was resuspended in 20 mM Tris-HCl buffer (pH 7.6) that contained DNase I (Sigma) and a protease inhibitor cocktail (Roche). The cells were then disrupted by passing them through a French press three times at 1,000 psi (6.89 MPa) of pressure. The crude extract was centrifuged at 200,000 × *g* for 1 h at 4°C (Beckman Coulter Optima LE-80K), and supernatant containing the soluble protein fraction was loaded into a Q-Sepharose column, previously equilibrated with 20 mM Tris-HCl buffer (pH 7.6). A gradient from 0 to 1 M NaCl in the same buffer was applied, but PmcA did not bind to the column and was eluted in the flow-through during the wash step. This fraction was concentrated and loaded onto a SP-Sepharose column equilibrated with 20 mM Tris-HCl buffer (pH 7.6). The fraction containing PmcA was eluted at 100 mM NaCl, using a gradient from 0 to 1 M NaCl in 20 mM Tris-HCl buffer (pH 7.6). All chromatography fractions were analyzed by SDS-PAGE (12% gel) stained for heme proteins (55) and by UV-visible spectroscopy to select those containing pure protein, measured by the A_Soret peak_/A_280nm_ ratio. The identity of PmcA was confirmed by N-terminal sequencing and mass spectrometry.

#### Production of STC and FccA from *Shewanella oneidensis*

STC and FccA proteins were produced and purified, as described in reference 37. Fractions containing pure protein were analyzed by SDS-PAGE (12% gel) and UV–visible spectroscopy. Pure protein has a typical absorbance ratio, A_Soret peak_/A_280nm_, larger than 4.5.

#### Protein preparation for *in vitro* experiments

The final purified proteins were washed and concentrated at 4°C using Amicon Ultra Centrifugal Filters (Millipore) with 5 (MtoD and PmcA), 30 (MtoA), and 50 kDa (ImoA) cutoff. Proteins were prepared in 20 mM potassium phosphate buffer (pH 7.6) containing 300 mM NaCl. For experiments involving ImoA, 0.05% DDM was included in the buffer to prevent protein precipitation during assays. Protein concentration was estimated using the absorption coefficient, ε_409nm_, of 125,000 M^−1^cm^−1^ per heme for the oxidized state of the protein (56).

### Spectroscopic techniques

#### UV-visible spectroscopy

UV-visible spectra of the proteins were acquired in potassium phosphate buffer (7.6) on a Shimadzu UV-1800 spectrophotometer (Shimadzu, Gamby, OR, USA) in the 250 to 800 nm wavelength range at room temperature. Spectra in the reduced state were obtained by the addition of small volumes of concentrated sodium dithionite solution (Sigma) to the oxidized sample.

#### NMR spectroscopy

NMR experiments were performed at 25°C on an NEO 500 MHz NMR spectrometer (Bruker, Rheinstetten, Germany) equipped with a 5 mm TCI C/N Prodigy Cryo probe. Then, 10% (v/v) of ^2^H_2_O (99.9 atom %) was added to the protein sample before spectral acquisition. The ^1^H-1D-NMR spectrum was acquired with a spectral width of 40 kHz and processed in the Topspin 4.1.1 software from Bruker using an exponential apodization function. Chemical shifts are reported in parts per million (ppm), and the proton spectrum was calibrated using the water signal as the internal reference.

For the ^1^H-1D-NMR stock samples of the proteins STC and FccA in 20 mM potassium phosphate buffer (pH 7.6) with 300 mM NaCl and 0.05% DDM were lyophilized and dissolved in ^2^H_2_O (99.9 atom %). For all the other proteins, 10% (v/v) of ^2^H_2_O (99.9 atom %) was added to the protein sample before spectral acquisition. To study the interactions, protein samples containing 50 µM or 100 µM were titrated against increasing concentrations of another cytochrome. ^1^H-1D-NMR spectra were recorded before and after each addition. The relative ratio used in the protein interaction studies was chosen based on the presence or absence of observable shifts in the spectral signals (57, 58). Chemical shifts are reported in ppm, and the proton spectrum was calibrated using the water signal as the internal reference.

#### RR spectroscopy of ImoA

RR spectra were acquired using a confocal Raman spectrometer (Jobin-Yvon LabRam 800 HR, HORIBA) equipped with a liquid nitrogen-cooled CCD detector, employing 406 nm excitation from a krypton ion laser (Coherent INNOVA 300c). Spectra were measured from 100 µL of 100 µM of ImoA placed into a rotating cuvette (Hellma) to prevent prolonged exposure of individual protein molecules to laser irradiation. Spectra were recorded with 0.8-mW laser power and 20-s accumulation time at room temperature; eight spectra were co-added in order to improve signal-to-noise ratio. Spectral deconvolution was performed using LabSpec 5.4 software.

#### EPR spectroscopy of PmcA

X-band EPR spectrum was obtained using a Bruker EMX spectrometer equipped with an Oxford Instruments ESR-900 continuous flow helium cryostat (Oxford Instruments, Oxfordshire, UK), and a high-sensitivity perpendicular-mode rectangular cavity. An as-purified solution of PmcA was prepared in 20 mM potassium phosphate buffer (pH 7.6) containing 300 mM NaCl to a final concentration of 100 µM. The spectrum was recorded at 15K using a microwave frequency of 9.39 GHz, a modulation amplitude of 1.0 mT, and a microwave power of 2 mW. Spectra were analyzed and simulated using SpinCount (59).

### Cyclic voltammetry

Protein film voltammetry was carried out at 25°C using a three-electrode electrochemical cell configuration consisting of a PGE working electrode (IJ Cambria Scientific), an Ag/AgCl (3 M KCl) reference electrode, and a graphite rod counter electrode. The experiments were performed inside a Coy anaerobic chamber using an electrochemical analyzer from CHI Instruments controlled by the manufacturer’s software (version 20.04). The PGE electrode was cleaned and freshly polished before every experiment. For these experiments, 3 μL of pure protein (ImoA or PmcA) was deposited on the surface of the working electrode and left to dry for approximately 30 min. Excess and/or unattached proteins were removed by rinsing the electrode with distilled water. For ImoA and PmcA, the electrode was immersed in 3 mL of a mixed buffer solution containing 5 mM of HEPES (Sigma Aldrich), 5 mM of MES (Sigma Aldrich), 5 mM of TAPS (Sigma Aldrich), and 100 mM KCl. The desired pH values were adjusted with 1 M NaOH. Voltammograms were acquired at a scan rate of 0.4 Vs^−1^ for ImoA and PmcA. QSoas program (version 1.0) available at https://bip.cnrs.fr/groups/bip06/software/ (60) was used to subtract the capacitive current and suppress background noise of the raw electrochemical data. All potentials are reported with respect to SHE by addition of 197 mV (61) to those measured.

### Bioelectrochemical experiments

To evaluate the ability of the different *Shewanella* strains carrying different genes from ES-1 in performing EET to anodes, the strains were cultivated in a three-electrode single-chambered bioelectrochemical reactor (33). Briefly, the reactors were made out of 100-mL Schott glasses closed with butyl rubber and fixed by screw caps to guarantee anoxic conditions during the experiment. The working electrode was made of graphite felt (GFD 2.5 from Sigracell (Germany), round size of 12 mm diameter), while the counter electrode was a graphite rod. Both electrodes were connected with titanium wires. The reference electrode (IJ Cambria) used was Ag/AgCl (3 M KCl). The total surface area of the anode electrode in contact with the medium was 2.26 cm^2^. Prior to autoclaving, the working electrode was immersed in isopropanol and washed with deionized water. Before use, the reactors were filled with deionized water and autoclaved. Bacteria were grown overnight in oxic LB medium containing 50 µg/mL kanamycin, then washed once with *Shewanella* Basal Medium (SBM) (62) and inoculated into fresh SBM containing 20 mM sodium lactate and 50 µg/mL kanamycin, adjusted to an OD_600nm_ of approximately 9–10. After the current had stabilized, the reactors were inoculated with washed *Shewanella* cells at a dilution of 1:100. All the experiments were performed at 30°C using a MultiPalmSens4 multipotentiostat (PalmSens, The Netherlands) in the chronoamperometric mode, applying 200 mV vs Ag/AgCl and measuring the current every 60 s. Bioelectrochemical experiments were conducted in triplicate for each bacterial strain and across biological replicates, with the strains' relative behavior consistent across duplicates and showing the same trends as those reported in the manuscript. Although the same strains, media, and operational conditions (temperature, stirring, and applied potential) were used, experiments were conducted on different days, and small variations in absolute current values are expected due to minor differences in inoculum and media batches. Consequently, analyses were restricted to comparisons within the same experiment.

### Model building using AlphaFold

The algorithm for protein structure prediction (AlphaFold3) (63) was used to model the three-dimensional structure of CymA, ImoA and PmcA, including the appropriate number of heme cofactors. The signal peptide of PmcA was cut using Signal P 5.0 (64). The predicted structures were processed and aligned using PyMOL (65).

## SUPPLEMENTAL MATERIAL

Supplemental material aem.02536-25-s0001.pdf Tables S1 to S3; Fig. S1 to S14.

ASM does not own the copyrights to Supplemental Material that may be linked to, or accessed through, an article. The authors have granted ASM a non-exclusive, world-wide license to publish the Supplemental Material files. Please contact the corresponding author directly for reuse.
