# Supplementary material for: Deciphering the Mto electron uptake pathway of Sideroxydans lithotrophicus ES-1
Source: Appl Environ Microbiol. 2026 Jun 2;92(7):e02536-25. doi: 10.1128/aem.02536-25 (PMC13390432; doi:10.1128/aem.02536-25)
Supplement: Supplemental material — Tables S1 to S3; Fig. S1 to S14. [file aem.02536-25-s0001.pdf]

Deciphering the Mto electron uptake pathway in *Sideroxydans lithotrophicus* ES-1

Anaísa Coelho[a,#], Büşra Bayar[a], Bruno M. Fonseca[a], Abhiney Jain[b], Smilja Todorovic[a], Filipe Folgosa[a], Jeffrey A. Gralnick[b], Ricardo O. Louro[a], Catarina M. Paquete[a*]

Author's affiliation:

[a] Instituto de Tecnologia Química e Biológica António Xavier, Universidade Nova de Lisboa, Av. da República, 2780-157 Oeiras, Portugal

[b] BioTechnology Institute and Department of Plant and Microbial Biology, University of Minnesota — Twin Cities, St. Paul, MN 55108, USA

* For manuscript correspondence: cpaquete@itqb.unl.pt

[#] Currently at Department of Physics and Astronomy, University of Southern California, Los Angeles, CA 90089, USA

Table S1. Proteins known to be involved in ubiquinone and menaquinone synthesis in bacteria.

Protein	Presence/absence on ES-1 genome	Function
UbiA	+	Ubiquinone synthesis
UbiD	+	Ubiquinone synthesis
UbiX	+	Ubiquinone synthesis
UbiI	+	Ubiquinone synthesis
UbiG	+	Ubiquinone synthesis
UbiH	+	Ubiquinone synthesis
UbiE	+	Ubiquinone synthesis
UbiF	+	Ubiquinone synthesis
XanB2	+	Ubiquinone synthesis
MenA	+	Ubiquinone synthesis
MenB	-	Menaquinone synthesis
MenC	-	Menaquinone synthesis
MenD	-	Menaquinone synthesis
MenE	-	Menaquinone synthesis
MenF	+	Menaquinone synthesis
MenH	-	Menaquinone synthesis

Table S2. Paralogues of MtoD encoded on *S. lithotrophicus* ES-1 genome.

**Locus Tag**	**Percent identity to MtoD protein sequence**
Slit_2693	33%
Slit_1770	36%
Slit_0019	28%
Slit_2780	37%
Slit_0510	38%
Slit_2042	30%
Slit_1353	31%

Table S3. Plasmids and primers used in this study.

Plasmids, primers	Relevant characteristics	Source
Plasmids		
pBBR1MCS-2	Broad range cloning vector, Km^r	(1)
pBAD202/D-TOPO	Expression vector, Km^r	Invitrogen
pBAD202::*imoA*	pBAD202/D-TOPO containing ImoA and a Strep-tag sequence.	This study
pBAD202::*pmcA*	pBAD202/D-TOPO containing PmcA	This study
pBAD202::*sp-ompA_mtoD*	pBAD202/D-TOPO containing MtoD with the signal peptide of OmpA and a Strep-tag sequence	(2)
pBAD202::*sp-ompA_mtoA*	pBAD202/D-TOPO containing MtoA with the signal peptide of OmpA and a Strep-tag sequence.	This study
pBBR1MCS-2::*imoA*	pBBR1MCS-2 containing ImoA	This study
pBBR1MCS-2::*mtoD*	pBBR1MCS-2 containing MtoD	This study
pBBR1MCS-2::*pmcA*	pBBR1MCS-2 containing PmcA	This study
pBBR1MCS-2::*imoA_pmcA*	pBBR1MCS-2 containing ImoA and PmcA	This study
pBBR1MCS-2::*imoA_mtoD*	pBBR1MCS-2 containing ImoA and MtoD	This study
Primers	***Sequence (5' to 3')***	
*fccA*_UP_F	NNACTAGTTGCAGCGGTGCTATTAA	(3)
*fccA*_UP_R	NNGAATTCCATTGCGCCAGAGATCA	(3)
*fccA*_DN_F	NNGAATTCATCGCGGGTGCATCTGC	(3)
*fccA*_DN_R	NNGAGCTCATGGCAGGCTGATAGGC	(3)
pBAD_ImoA_F	GAAGGAGATATACATACCATGATAACAAGACTG GCATCCTGAAACGTTC	This study
pBAD_ImoA_R	TTTATCAGATCCCATGTCACTTCTCGAACTGCGGA TGGCTCCAGGGCCCCTGGAACAGAACTTCCAGCT CCTCATCCGGATCGCGGTAC	This study

pBAD_PmcA_F	GAGATATACATACCATGAAAAAGACAGCTATCG CGATTGC	This study
pBAD_PmcA_R	TATCAGATCCCATGCTACTTCTCGAATTGTGGAT GAGAC	This study
pBAD_MtoA_F	GAGATATACATACCATGAAAAAGACAGCTATCG	This study
OmpA_MtoA_F	CTGCAGGCTGCTCATCTGCTGCCTGCGCAACGGT AGC	This study
OmpA_MtoA_R	GCTACCGTTGCGCAGGCAGCAGATGAGCAGCCT GCAG	This study
pBAD_MtoA_R	TATCAGATCCCATGCTACTTCTCGAATTGTGGAT GAGACCAGCGTTGCAAGTAGCCGCC	This study
pBBR_ImoA_F	CCCCCCCTCGAGATGAATAACAAGACTGGC	This study
pBBR_ImoA_R	ATGTTTTTCCTCCTACTAGTTCACTCCTCATCCG	This study
pBBR_PmcA_F	GGCCCCCCCTCGAGATGAAAAACCAGACATTGC TAAAAGGCATTGTTGTCGCTGTGTCACTGTATGG AATGGCTTTTACTCCGATGGCATTCGCGGATGTC AGTTTCAAAAG	This study
pBBR_PmcA_R	CTCTAGAACTAGTGGATCCTCAGTTGTTCTTCGC C	This study
pBBR_MtoD_F	CCCCCCCTCGAGAGGAGGAAAAACATATGACTC G	This study
pBBR_MtoD_R	CCGCGGTGGAGCTCCTAGAGCGAAAGGATCC	This study

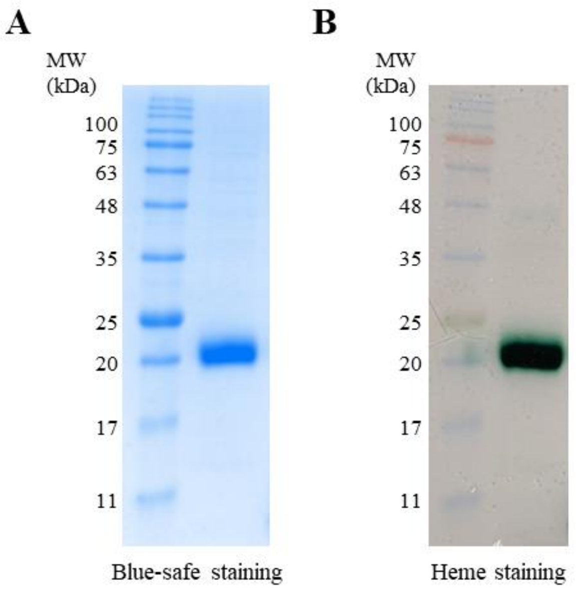

Figure S1. SDS-PAGE gel of ImoA. Gel stained for **(A)** Blue-safe and **(B)** hemes.

```
ImoA      ---MNNKTGILKRSWDFLKQPSAKYSLLTLLVVGFFSGIIFWGGFNTGMEATNRLEFCIGCH    59
NapC      MGNSDRKPGLIKRLWKWWRTPS-RLALGTLLLIGFVGGIVFWGGFNTGMEKANTEEFCISCH    61
CymA      ------------MNWRALFKPSAKYSILALLVVGIVIGVVGYFATQQTLHATSTDAFCMSCH    50
NrfH      ------------MSE---EKSRNGPARLKLVLGGATLGVVALATVAFGMKYTDQRPFCTSCH    47
                                   :   *:: *   *::        :. :.   ** .**

ImoA      EMRDNVYQEYKKTIHYANRTGVRAICSDCHVPKDWTHKLIRKIQA-SQEIWGKLTGY-VDTP    119
NapC      EMRNTVYQEYMDSVHYNNRSGVRATCPDCHVPHEFVPKMIRKLKA-SKELYGKIFGV-IDTP    121
CymA      SNH-SLKNEVLASAHGGGKAGVTVQCQDCHLPHGPVDYLIKKIIV-SKDLYGFLTIDGFNTQ    110
NrfH      IMNPVGVT------HKLS-GHANISCNDCHAPHNLLAKLPFKAIAGARDVYMNTLGH----P    98
            .           *  .   .   * *** *:     :  *  . :::::

ImoA      EKFESHRMELATHEWARMKASDSRECRNCHSFDAMSGDIQ-KQTVYNKH----MKARADGQT    176
NapC      QKFEAHRLTMAQNEWRRMKDNNSQECRNCHNFEYMDTTAQ-KSVAAKMH----DQAVKDGQT    178
CymA      AWLDENRKEQADKALAYFRGNDSANCQHCHTRIYENQPETMKPMAVRMHTNNFKKDPETRKT    172
NrfH      GDLILAGM--------ETKEVVNANCKACHTMTNVEV-----------------ASMEAKKY    135
            :               :   . :*: **.    .                        :

ImoA      CIDCHKGIAHHLPKEYRD---PDEE    198
NapC      CIDCHKGIAHKLPDMREV---EPGF    200
CymA      CVDCHKGVAHPYPKG----------    187
NrfH      CTDCHRNVQHMRMKPISTREVADE-    159
          * ***:.: *   .
```

Figure S2. Multiple sequence alignment of NapC/NirT family proteins. Sequence alignment of *Sideroxydans lithotrophicus* ES-1 ImoA, *Escherichia coli* NapC, *Shewanella oneidensis* MR-1 CymA and *Desulfovibrio vulgaris* NrfH using ClustalW. The heme binding motifs are shown in red boxes. The distal histidine of the low-spin hemes are highlighted in blue. The methionine residue conserved in some NapC/NirT family proteins is highlighted in purple. Key residues in the putative quinone binding site are shown in green.

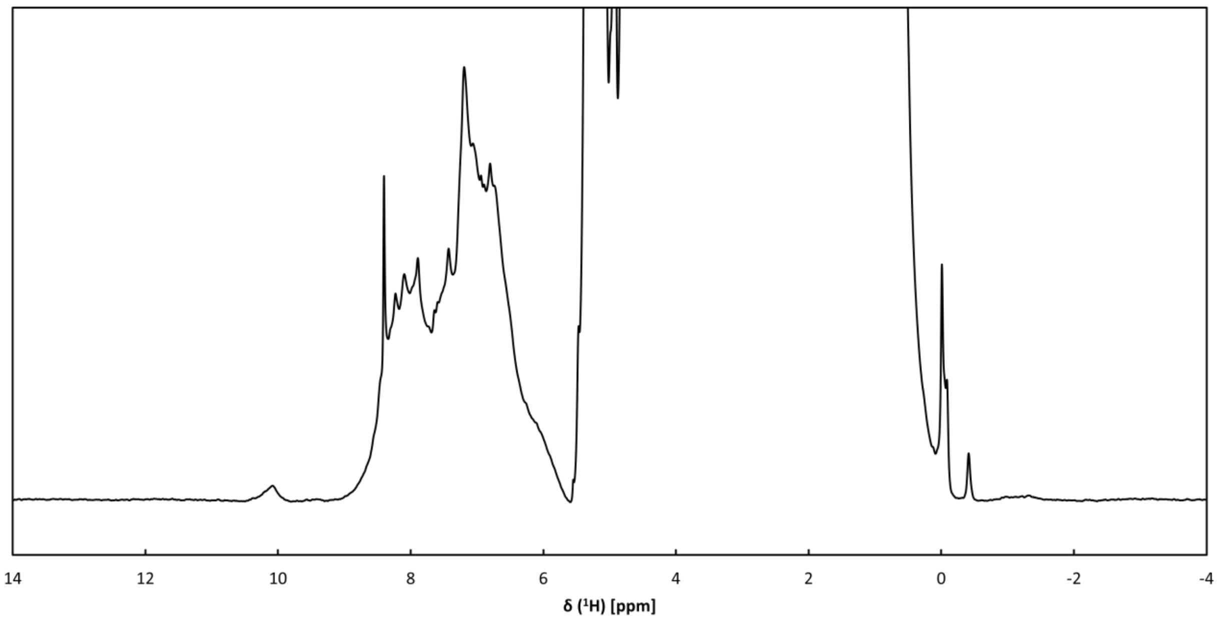

Figure S3. ^{1}H 1D NMR spectrum of reduced ImoA. The lack of the -3 ppm upfield signal indicates that a methionine does not axially coordinate the hemes.

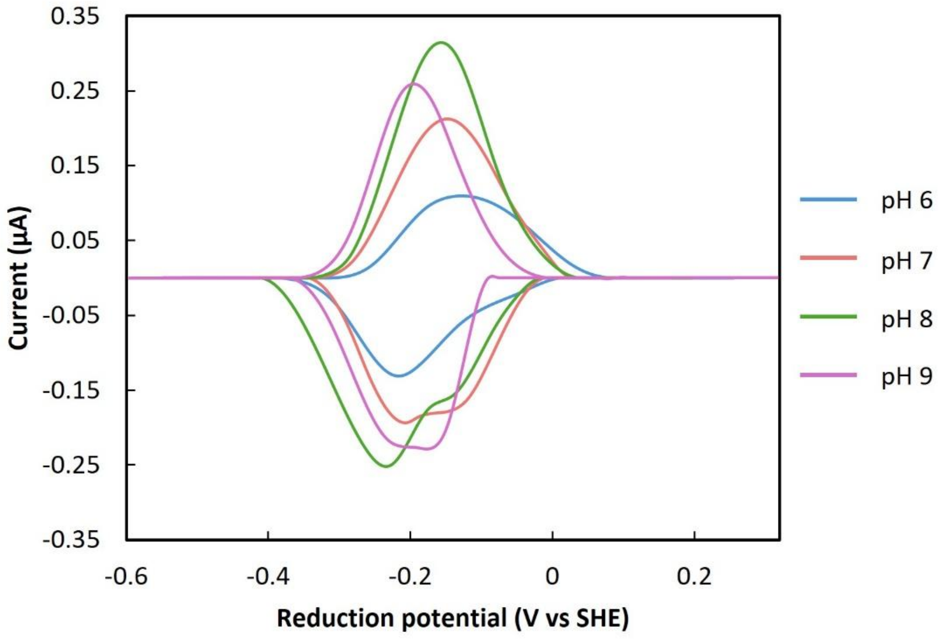

Figure S4. Cyclic voltammetry of ImoA. Baseline-subtracted data of the voltammograms obtained at a scan rate of 400 mV/s at different pH values.

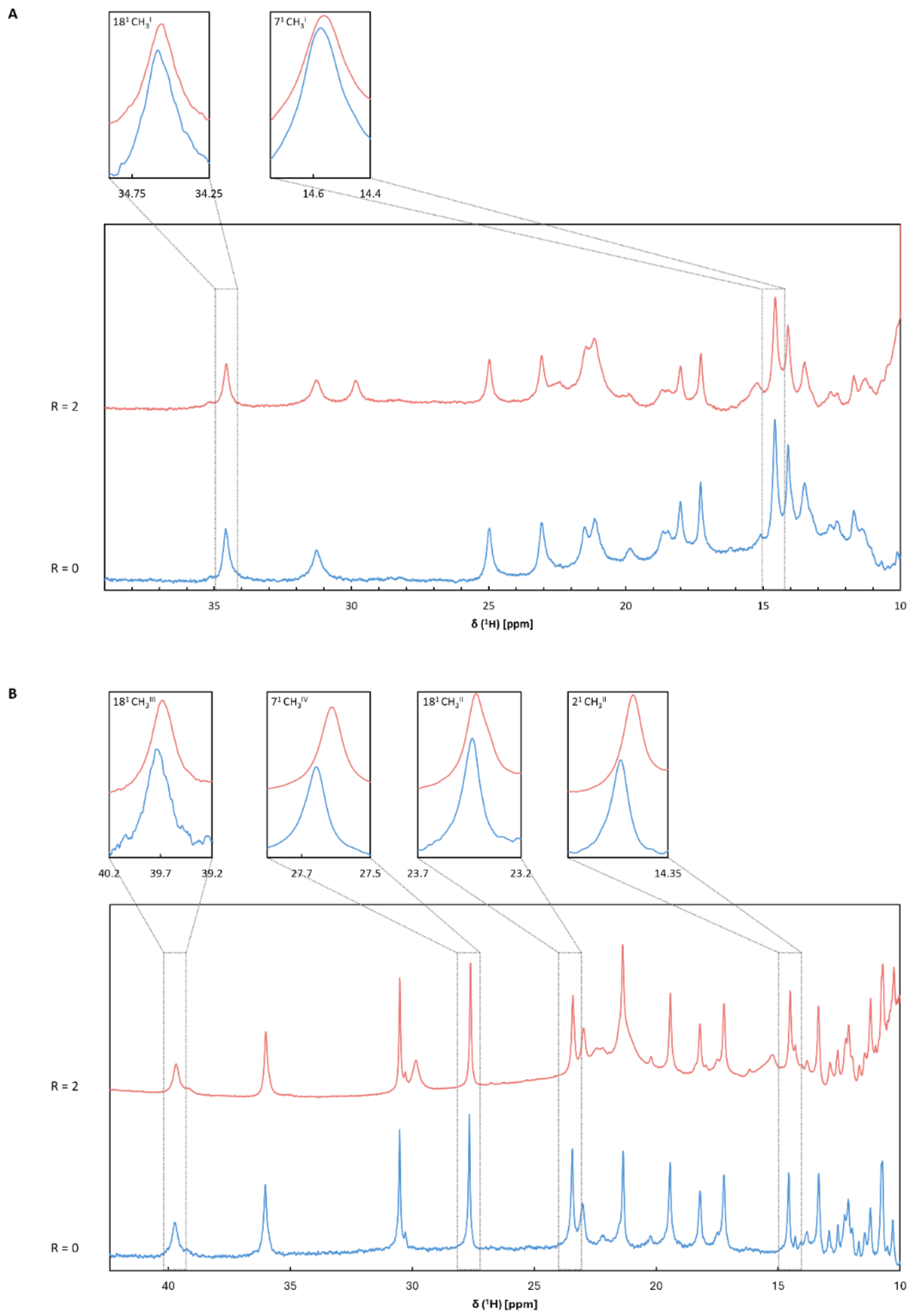

Figure S5. 1D ^{1}H NMR spectral changes of FccA (A) and STC (B) in the presence of ImoA. The bottom spectra were obtained with the individual proteins, while the top spectra were obtained upon addition of ImoA (molar ratio of [ImoA]/[STC or FccA] = 2). Boxes show examples of methyl signals with significant chemical shift perturbation. The methyl group is identified using the IUPAC-IUB nomenclature for hemes. The Roman numeral corresponds to the order of heme binding to the polypeptide chain.

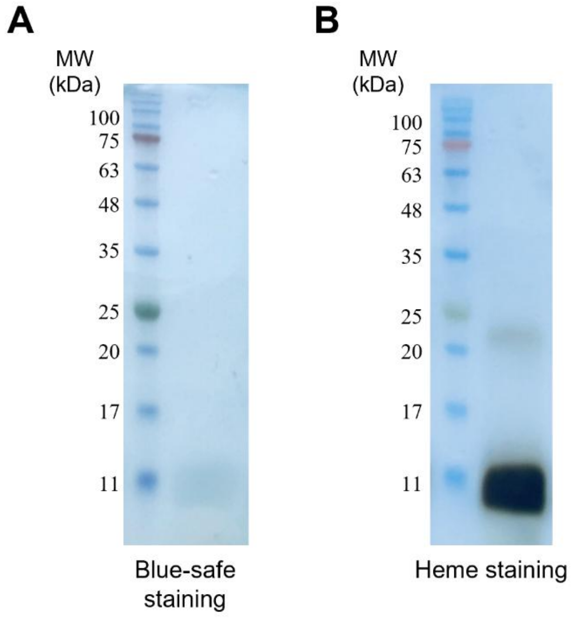

Figure S6. SDS-PAGE gel of PmcA. Gel stained for **(A)** Blue-safe and **(B)** hemes.

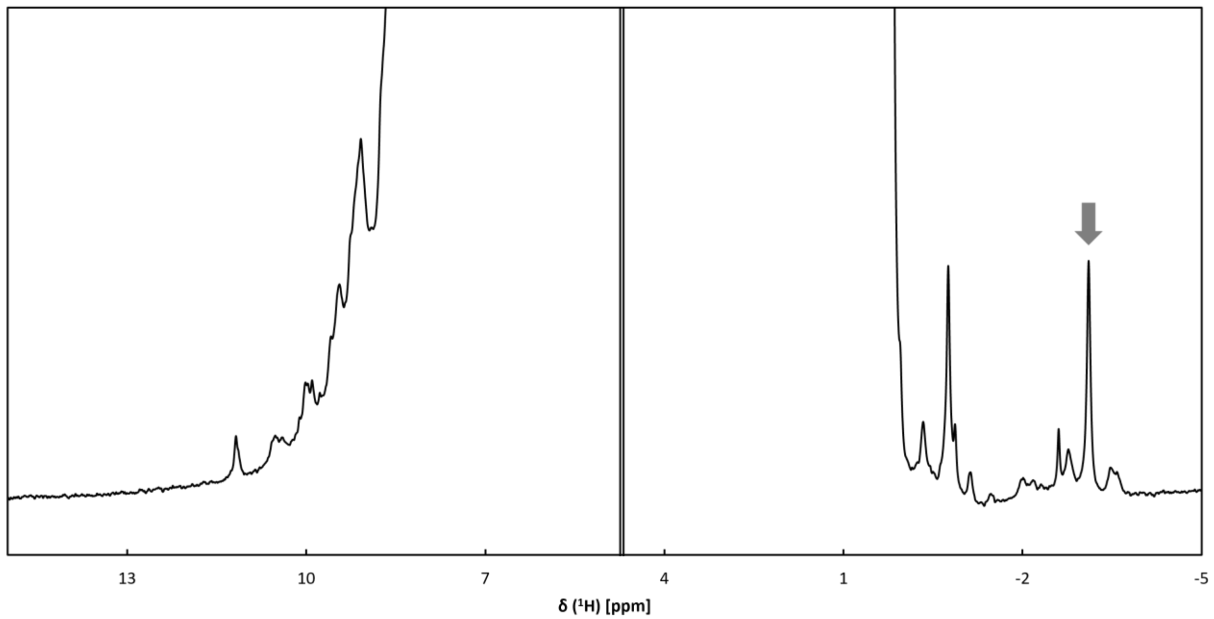

Figure S7. ^{1}H-1D-NMR spectrum of reduced PmcA. The -3 ppm upfield signal indicating that the heme is axially coordinated by a methionine is shown by an arrow.

A

```
PmcA    DVSFKSQVKPIIHDYCLSCHQPGGQGYEKSGLDMRSYKSLMKGTKFGSIIKPGDSFSSIM   60
MtoD    AVDVDAAKSLARENNCFKCHGVDK---EKDGPSYKKVAEKYRGKA--------DAEAKLI   49
         *...:  .   .: *:.**  .    **.* . :.  .  :*.         *: :.::

PmcA    IQLVEGRAHASIKMPFGT--------GGLAKDKVDVLKTWVDQGAKNN   100
MtoD    HHVTSGE---KAKFPDGHEEEHKNINGKASPEAIKNLVDWILSL----   90
         ::..*.   . *:* *         *  : : :. *  *: .
```

B

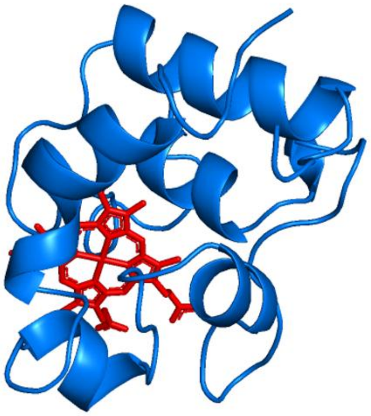

C

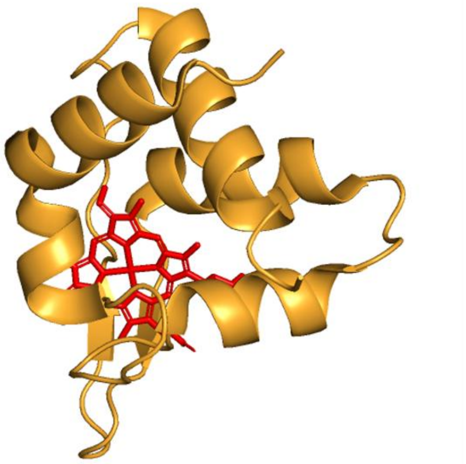

Figure S8. Comparison of PmcA and MtoD. (A) Sequence alignment of PmcA and MtoD using ClustalW. The heme binding motifs are shown in a red box, and the distal axial ligands of the heme are highlighted in blue. **(B)** Predicted three-dimensional structure of PmcA. **(C)** Three-dimensional structure of MtoD (4). The hemes in both proteins are colored in red.

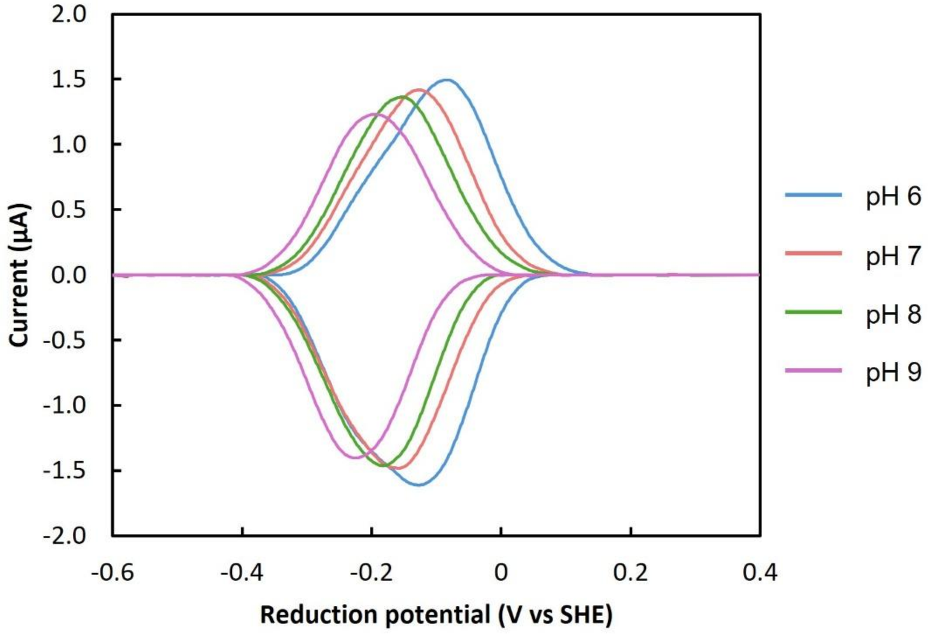

Figure S9. Cyclic voltammetry of PmcA. Baseline-subtracted data of the voltammograms obtained at a scan rate of 400 mV/s at different pH values.

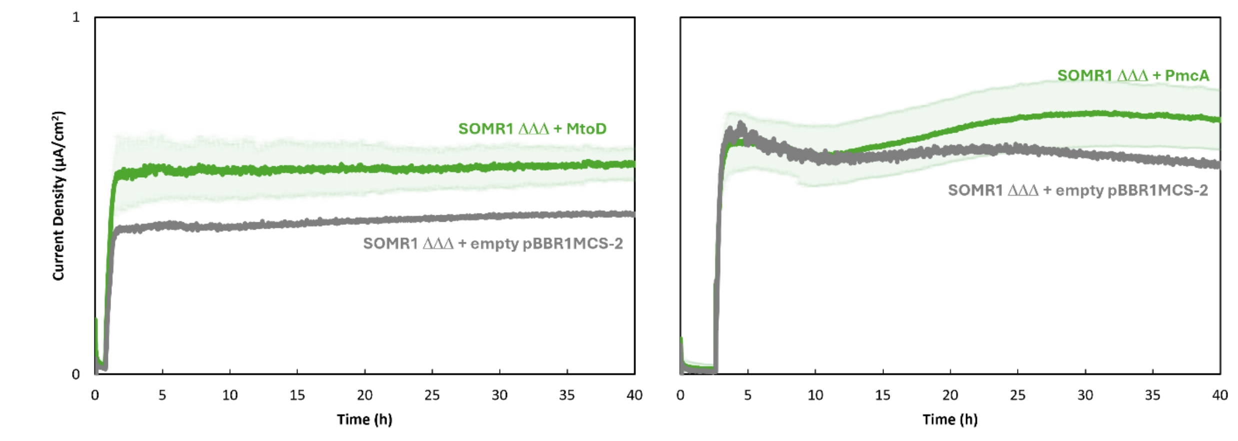

Figure S10. Current density produced by the different Δ*cymA* Δ*fccA* Δ*cctA* *Shewanella* strains. *Shewanella* Δ*cymA* Δ*fccA* Δ*cctA* strains containing pBBR1MCS-2::mtoD or pBBR1MCS-2::pmcA (green line), and empty pBBR1MCS-2 plasmid (grey line) in bioelectrochemical reactors. Error bars shown in the same colors represent the standard deviation of the mean from experiments performed in triplicate. Data for the *Shewanella* Δ*cymA* Δ*fccA* Δ*cctA* strains carrying empty pBBR1MCS-2 were obtained from a single reactor within the same experimental set.

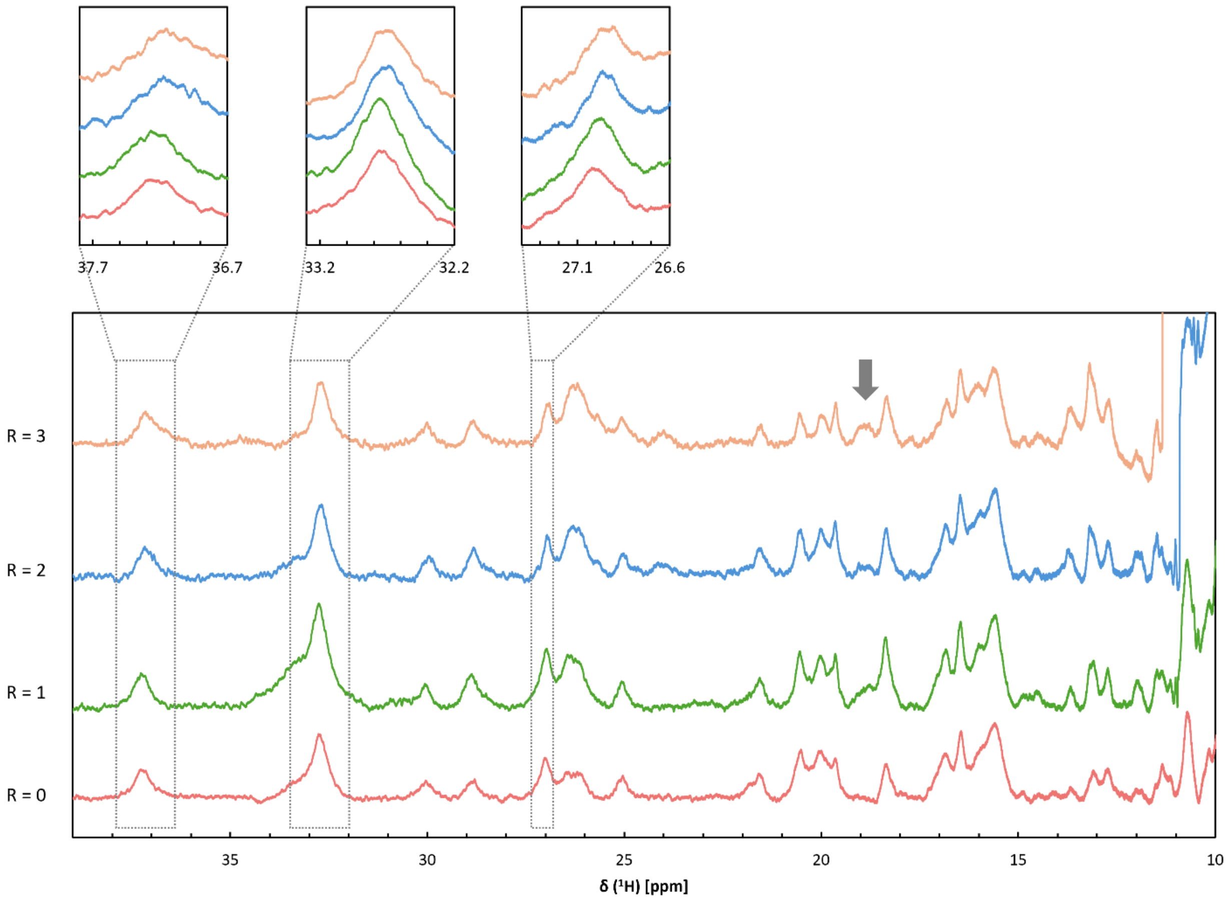

Figure S11. ^{1}H-1D-NMR spectral changes of MtoA in the presence of PmcA. The bottom spectrum was obtained with the individual protein MtoA in the oxidized state, while the top spectra were obtained upon increasing amounts of PmcA (molar ratio of [PmcA]/[MtoA]). Grey boxes indicate spectral changes in the MtoA cytochrome upon addition of the PmcA protein, while arrows highlight features that shift in the spectrum of the added cytochrome.

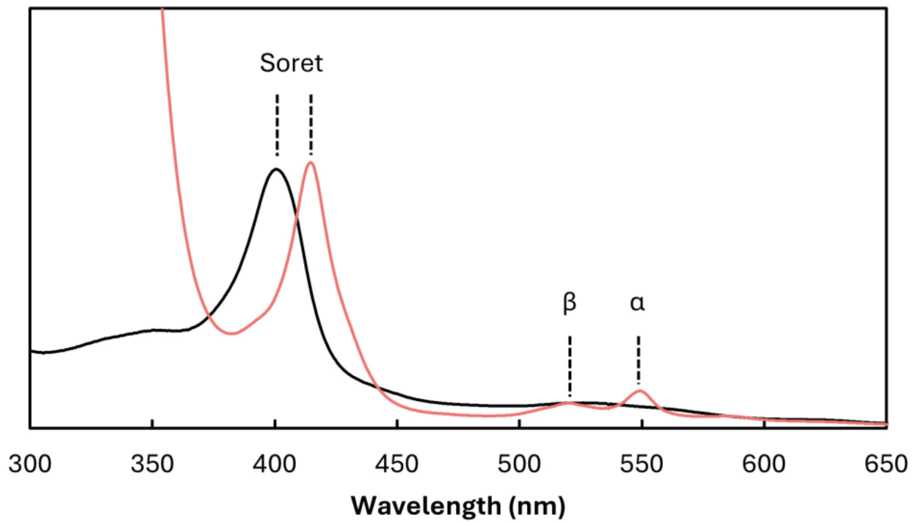

Figure S12. UV-visible spectra of purified MtoD. UV-visible spectra obtained in the oxidized (black) and reduced state (red line).

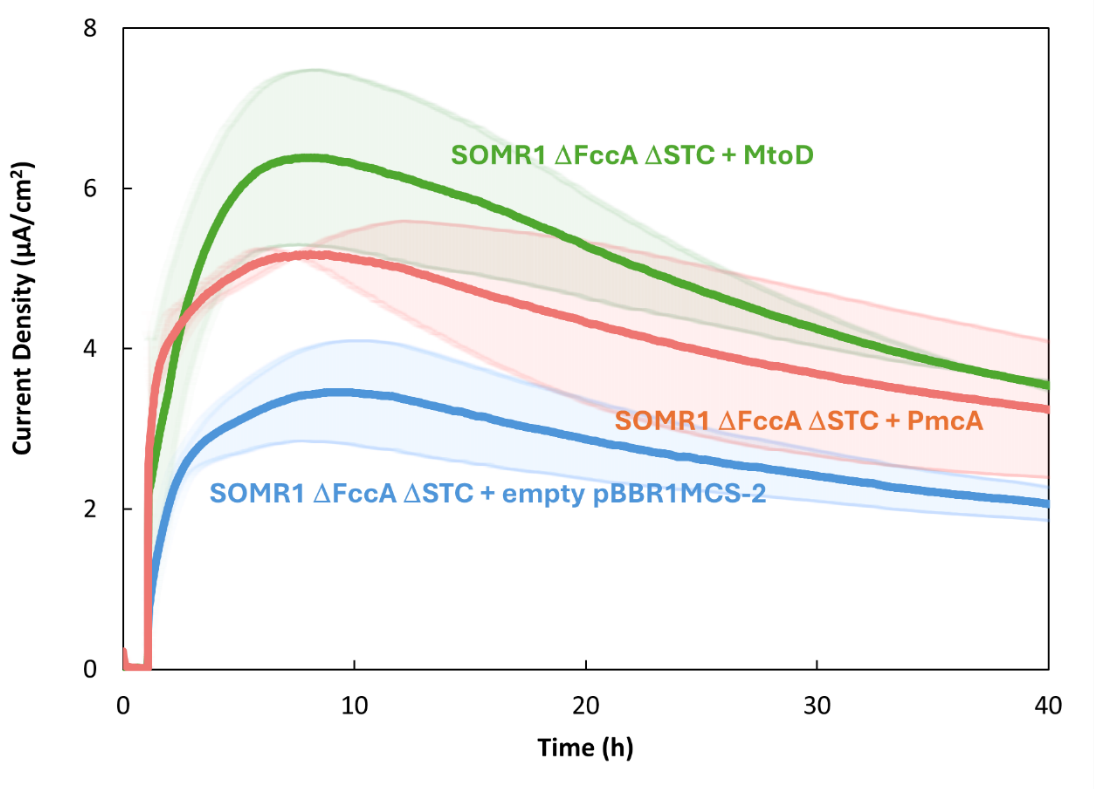

Figure S13. Current density produced by the different Δ*fccA* Δ*cctA* *Shewanella* strains. *Shewanella* Δ*fccA* Δ*cctA* strains containing pBBR1MCS-2 (blue line), pBBR1MCS-2::*mtoD* (green line), pBBR1MCS-2::*pmcA* (red line), in a bioelectrochemical reactor. Error bars shown in the same colors represent the standard deviation of the mean from experiments performed in triplicate for *Shewanella* Δ*fccA* Δ*cctA* strains containing pBBR1MCS-2, and in duplicate for *Shewanella* Δ*fccA* Δ*cctA* strains containing pBBR1MCS-2::*mtoD*, and pBBR1MCS-2::*pmcA*.

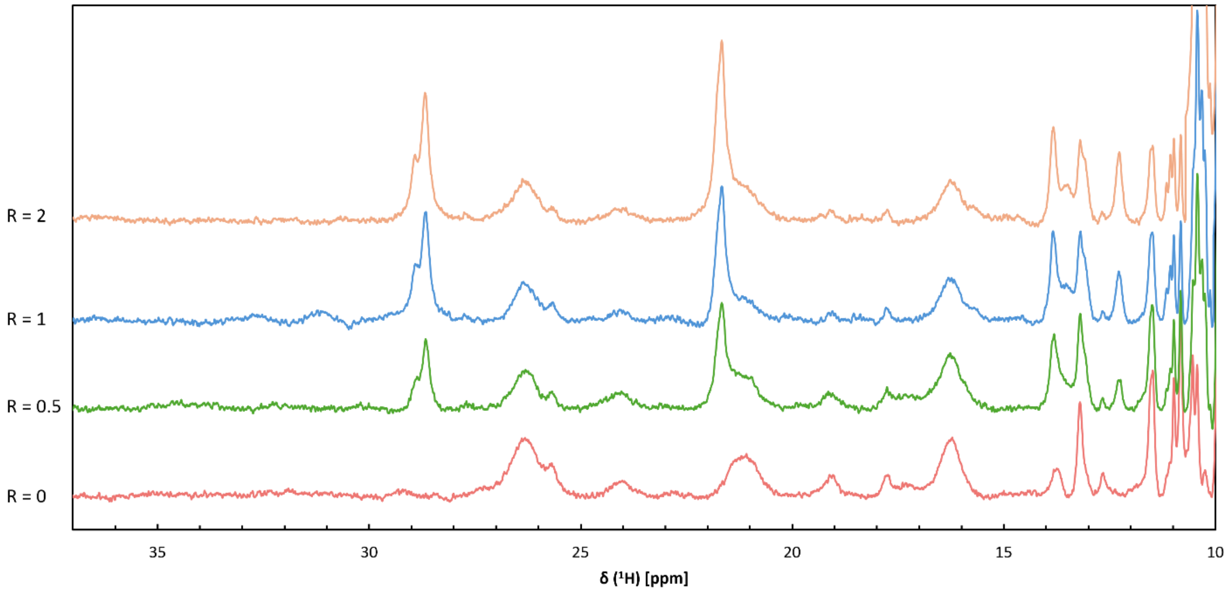

Figure S14. ^{1}H-1D-NMR spectral changes of PmcA in the presence of MtoD. The bottom spectra were obtained with PmcA in the oxidized state, while the top spectra were obtained upon increasing amounts of MtoD (molar ratio of [MtoD]/[PmcA]).

References:

1. Kovach ME, Elzer PH, Steven Hill D, Robertson GT, Farris MA, Roop RMM, Peterson KM, Hill DS, Robertson GT, Farris MA, Roop RMM, Peterson KM. 1995. Four new derivatives of the broad-host-range cloning vector pBBR1MCS, carrying different antibiotic-resistance cassettes. Gene 166:175–176.
2. Coelho A, Silva JM, Cantini F, Piccioli M, Louro RO, Paquete CM. 2024. Resonance assignments of cytochrome MtoD from the extracellular electron uptake pathway of *Sideroxydans lithotrophicus* ES-1. Biomol NMR Assign 18:139–146.
3. Ross DE, Flynn JM, Baron D, Gralnick J, Bond DR. 2011. Towards electrosynthesis in *Shewanella*: energetics of reversing the Mtr pathway for reductive metabolism. PLoS One 6:e16649.
4. Beckwith CR, Edwards MJ, Lawes M, Shi L, Butt JN, Richardson DJ, Clarke TA. 2015. Characterization of MtoD from *Sideroxydans lithotrophicus*: A cytochrome c electron shuttle used in lithoautotrophic growth. Front Microbiol 6:332.
